# Link prediction using low-dimensional node embeddings: The measurement problem

**DOI:** 10.1073/pnas.2312527121

**Published:** 2024-02-16

**Authors:** Nicolas Menand, C. Seshadhri

**Affiliations:** ^a^Department of Computer and Information Science, University of Pennsylvania, Philadelphia, PA 19104; ^b^Department of Computer Science, University of California, Santa Cruz, CA 95064

**Keywords:** low-dimensional embeddings, link prediction, graph representational learning, graph embeddings, machine learning metrics

## Abstract

Graph representation learning is a fundamental technique for machine learning (ML) on complex networks. Given an input network, these methods represent the vertices by low-dimensional real-valued vectors. These vectors can be used for a multitude of downstream ML tasks. We study one of the most important such task, link prediction. Much of the recent literature on graph representation learning has shown remarkable success in link prediction. On closer investigation, we observe that the performance is measured by the AUC (area under the curve), which suffers biases. Since the ground truth in link prediction is sparse, we design a vertex-centric measure of performance, called the VCMPR@k plots. Under this measure, we show that link predictors using graph representations show poor scores. Despite having extremely high AUC scores, the predictors miss much of the ground truth. We identify a mathematical connection between this performance, the sparsity of the ground truth, and the low-dimensional geometry of the node embeddings. Under a formal theoretical framework, we prove that low-dimensional vectors cannot capture sparse ground truth using dot product similarities (the standard practice in the literature). Our results call into question existing results on link prediction and pose a significant scientific challenge for graph representation learning. The VCMPR plots identify specific scientific challenges for link prediction using low-dimensional node embeddings.

## Significance

Link prediction is a fundamental machine learning task on complex networks, used to evaluate the central technique of low-dimensional embeddings. Our results question the common wisdom that low-dimensional embeddings perform well in link prediction tasks. We show that this wisdom is based on faulty measurements (based on area under the curve) used to evaluate link prediction. We propose vertex-centric local measures, under which existing low-dimensional embedding methods are shown to fail in link prediction. We identify a mathematical connection between this poor performance and the low-dimensional geometry of the node embeddings. Under a formal theoretical framework, we prove that low-dimensional vectors cannot capture sparse ground truth using dot product similarities (which is the standard practice in the literature).

Measurement is central to any scientific endeavor. Informative measurements are crucial to guide experiments and interpret results. For machine learning (ML), measurements are central to evaluating performance and discovering better techniques. These measurements have a large impact in the deployment of real ML systems. As such systems become a large part of modern society, it becomes even more important to have sound measurements of ML methods.

Our focus is on the important field of graph machine learning, where ML is deployed on large complex networks. One of the recent advances in ML uses graph representation learning or low-dimensional node embeddings to tackle a large variety of tasks. The input is a graph G on n vertices. These methods map each vertex to a vector in $R^{d}$, where d is typically much smaller than n. (So n may be in millions or more, while d is typically 128.) These embeddings are generated in an unsupervised or self-supervised manner. The aim is to generate embeddings where geometric proximity (often measured as dot product) maps to graph proximity. The design of low-dimensional node embeddings is a popular and timely research area. We point the reader to surveys (1, 2) and Chapter 23 in ref. 3.

These embedding methods are evaluated by performing a downstream ML task, such as the classic link prediction problem (4, 5). Recall the formulation (4, 5). We are given a graph G=(V,E) as part of the training data. One should think of a dynamic process generating edges, and G is the current snapshot of edges. Our aim is to predict the future edges among previously seen nodes. The link predictor trains on the current snapshot G and does the following. Given a pair (i,j) of vertices, it predicts whether (i,j) will be an edge.

We identify a fundamental measurement problem. Most contemporary literature for link prediction using node embeddings measure performance by the AUC (area under the curve) metric (6–19). But the AUC is a measure that is meaningful for dense signal and suffers from imbalance biases (20, 21). We find that when link prediction performance is measured according to localized metrics, there is a significant drop in quality. This is a major scientific problem. AUC in link prediction is used to benchmark algorithms (11); used to evaluate new techniques (12); and conclude properties of ML algorithms (13). At least eight of the cited papers were published in the past 3 y (12–19), and two appear in extremely high profile scientific venues (12, 19). It is of central importance to have measurements that lead us to correct scientific conclusions.

Our paper investigates the connections between alternate vertex-centric link prediction measures, the sparsity of ground truth, and low-dimensional node embeddings.

## Our Results

We empirically demonstrate that the AUC metric for link prediction by node embedding methods leads to incorrect conclusions on the quality. We define vertex-centric measures, which clearly show poor performance on real-world datasets. These measures are used to construct VCMPR plots that quantitatively demonstrate the low quality on link prediction. To explain these results, we design a theoretical framework formally showing that commonly used link prediction algorithms from low-dimensional embeddings are unlikely to get high local precision/recall values. Inspired by these results, we pose a concrete challenge problem for low-dimensional embeddings. We hope that our measurements and challenge will inspire further research on this important topic.

## The VCMPR Scores and Empirical Setup

We train a large number of important node embedding methods on standard graph datasets (6–10, 18, 22, 23). We set up a transductive link prediction experiment, following the same setup as the above results. We are given a training graph $G=(V,E_{\mathrm{tr}})$. Based on this training data G, we design a predictor for future edges, denoted $\chi :V^{2}\to \left[0,1\right]$. One can think of the predictor as giving a score for each pair (i,j), which measures the likelihood that this pair will form an edge. The performance of χ is tested against another set $E_{\mathrm{test}}$ of edges. In practice, the edges $E_{\mathrm{tr}}$ and $E_{\mathrm{test}}$ are generated by randomly partitioning the edges of an existing dataset. Note that $E_{\mathrm{test}}$ is the ground truth for our experiment.

**Fig. 1.**
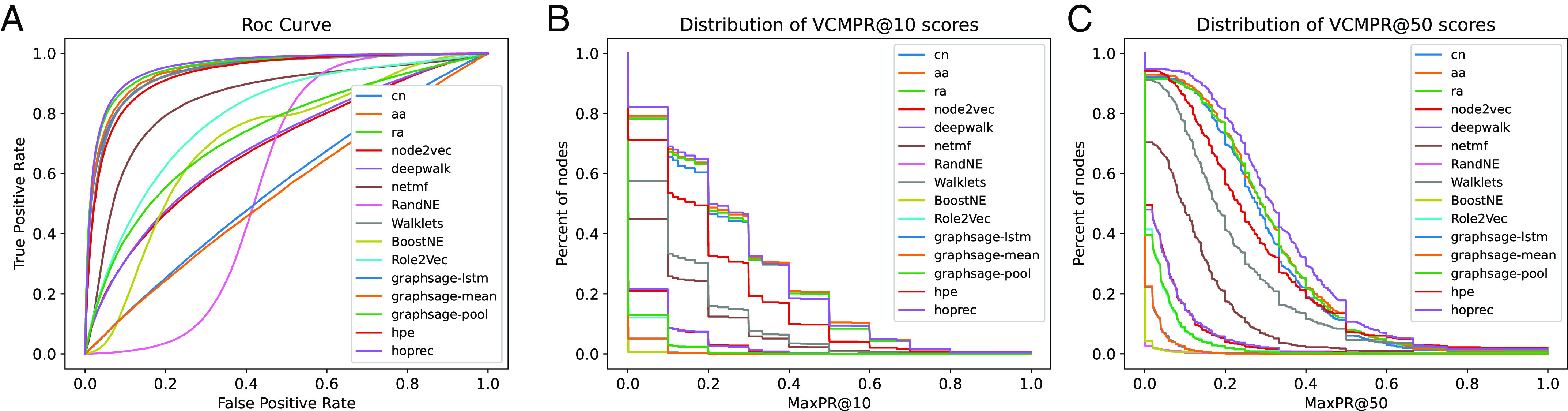
We train a number of important node embedding methods for transductive link prediction on the blog-Catalog dataset (25). (*A*) We plot the standard ROC curves for each predictor. (*B*) We plot the distribution of the VCMPR@10 values. (*C*) We plot the distribution of VCMPR@50 values. Observe that ROC curves are typically high leading to large AUC values. On the other hand, the VCMPR curves are quite low. This is indicative of both low precision and recall among the top predictions. The average values are summarized in Table 1.

We compute the standard ROC curve (receiver-operator characteristic) (24), as shown in Fig. 1*A* for Blog-Catalog dataset. Most methods perform quite well according to this plot. Similar results for the PR curve (Precision–Recall). These plots are often summarized using the “area under the curve” (AUC) metric, whose maximum value is one. We show the ROC-AUC and PR-AUC values in Table 1; consistent with the literature, we see AUC scores more than 0.7, and a largest score of 0.94. This would suggest good performance on transductive link prediction.

**Table 1.** This table complements Fig. 1, which has results from link prediction on the blog-Catalog dataset

	ROC AUC	PR AUC	Avg VCMPR @10	Avg VCMPR @50
Resource allocation	0.95	0.95	0.26	0.30
Adamic adar	0.95	0.94	0.26	0.30
Common neighbors	0.94	0.94	0.25	0.29
HOP-Rec	0.96	0.95	0.26	0.33
Walklets	0.94	0.93	0.12	0.22
HPE	0.93	0.93	0.19	0.27
NetMF	0.85	0.82	0.09	0.12
Role2Vec	0.79	0.76	0.01	0.03
GraphSage-MP	0.73	0.73	0.02	0.03
BoostNE	0.70	0.63	0.00	0.00
DeepWalk	0.69	0.69	0.03	0.05
Node2Vec	0.69	0.69	0.03	0.05
RandNE	0.58	0.49	0.00	0.00
GraphSage-L	0.56	0.55	0.01	0.01
GraphSage-M	0.54	0.53	0.01	0.01

We give the ROC-AUC, PR-AUC, and average VCMPR@k for k=10,50. We observe fairly large AUC values, with the largest being 0.96. But the average VCMPR@k values are quite low. For almost all methods, it is less than 0.2, which implies poor precision and recall at the individual vertex centric predictions.

For the same setup, we define the vertex-centric max precision recall at k (VCMPR@k) plots, a local metric. This measure requires a closer look into link prediction using node embeddings. For each pair of vertices (i,j), the classifier/predictor computes a score based on which it predicts an edge. For a given vertex i of nonzero degree $d_{i}$, we rank all other vertices j in decreasing order of their scores. Remove pairs from $E_{\mathrm{tr}}$. Take the top k scores from this list, and let $t_{i}\left(k\right)$ denote the number of ground truth edges in this list. Meaning, there are $t_{i}\left(k\right)$ ground truth edges (i,j) in the top k-entries of the (downward) sorted list.

$$\;\text{VCMPR@k for vertex}\;i=\frac{t_{i}\left(k\right)}{\min(k,d_{i})}.$$

Note that $t_{i}\left(k\right)/k$ is the precision@k, and $t_{i}\left(k\right)/d_{i}$ is the recall@k. We take the larger of these values, so that low degree vertices are not penalized for poor precision (since $t_{i}\left(k\right)\leq d_{i}$). Roughly speaking, if the VCMPR@k for vertex i is δ, then a δ fraction of the top-k predictions (for i) are actually ground truth edges. Note that VCMPR is a local metric and computed at a per-vertex basis. Contrast with the AUC, which is a global measure. We also stress the difference from vanilla precision/recall@k, which is computed by ranking (a sample of) all edges in the graph. (We discuss more differences and variants of VCMPR subsequently.) The VCMPR plots give the complementary cumulative histogram for the VCMPR values. So for x∈[0,1], we plot the fraction of vertices with VCMPR at least x. These are shown in Fig. 1*B* and *C*.

We observe that the VCMPR scores are surprisingly low. For example, even for the best method on this dataset, on average only 18% of the top scores are edges. And most methods have an average VCMPR of less than 0.05. In some datasets, the VCMPR values become a little higher (0.2 to 0.3), but nowhere near the large AUC values. We observe this consistently across methods and datasets (*Experimental Results*). Rather surprisingly, an AUC score of more than 0.7 may still lead to a VCMPR of less than 0.01. This means, for an average vertex, for the top (say) 50 predictions, at most one of them is a true edge. The difference between the high AUC and poor VCMPR is quite dramatic. The global “dense metric” of AUC is not attuned to the sparse ground truth. The VCMPR@k, for small k, is an averaged “local” score, and is more appropriate for sparse ground truth.

This measurement problem is apparent in the VCMPR plots of Fig. 1 *B* and *C*. The plots are quite low, as opposed to the high ROC curves in Fig. 1*A*. As an example, consider the Role2Vec algorithm (curve in pink). The VCMPR plots are almost at the bottom, despite the ROC curve being third from the *Top* in Fig. 1*A*. We see the ROC curves lead to misleading interpretations on prediction quality and should not be the basis for algorithm evaluation. We see identical issues with the PR-AUC.

## The Importance of Being Vertex-Centric

We stress the difference such as (vanilla) precision/recall@k, as well as the hits@k metric. The latter is used in knowledge completion tasks (26, 27). The hits@k metric is global. It takes a decreasing sorted list of random negative instances according to model score, and checks the number of test true positive edges that score in the top k. On the other hand, VCMPR@k scores are computed for every vertex. We stress that the k parameters for both metrics are incomparable. For example, consider the ogbl-collab dataset from the Open Graph Benchmark Leaderboard that comes with a specific link prediction task (26) The recommended metric is hits@50. For VCMPR, a natural choice of k is around the average vertex degree (which is 9). The average degree is the average length of a ground truth list, for a vertex.

In Table 2, we give the average AUC, VCMPR@10, and the recommended hits@50 metric scores for ogbl-collab dataset. The hits scores are significantly higher than the average VCMPR scores. HOP-REC, one of the embedding-based leaders on OGB, has a VCMPR@10 of just 0.29, while its hits score is 0.66. The hits score suggests reasonable performance, while the VCMPR scores are quite low. AUC scores are extremely high, as in Table 1. We get similar results on other examples, described in *SI Appendix*. Overall, the experiments show that the VCMPR and hits metrics are fundamentally different.

**Table 2.** This table complements Fig. 2, which has results from link prediction on the ogbl-collab dataset

	ROC-AUC	PR-AUC	Avg VCMPR @10	Hits @50
Common neighbors	0.83	0.91	0.26	0.53
Adamic adar	0.83	0.91	0.29	0.65
Resource allocation	0.83	0.91	0.29	0.65
DeepWalk	0.83	0.86	0.18	0.22
Node2Vec	0.83	0.86	0.19	0.22
NetMF	0.86	0.92	0.02	0.35
RandNE	0.72	0.73	0.12	0.09
Walklets	0.88	0.92	0.23	0.59
BoostNE	0.92	0.93	0.00	0.08
Role2Vec	0.90	0.93	0.16	0.44
GraphSage-M	0.51	0.51	0.00	0.00
GraphSage-MP	0.51	0.51	0.00	0.00
GraphSage-L	0.51	0.51	0.00	0.00
HPE	0.90	0.92	0.02	0.21
HOP-rec	0.97	0.98	0.29	0.66

We give the ROC-AUC, PR-AUC, average VCMPR@10 and Hits@50.

**Fig. 2.**
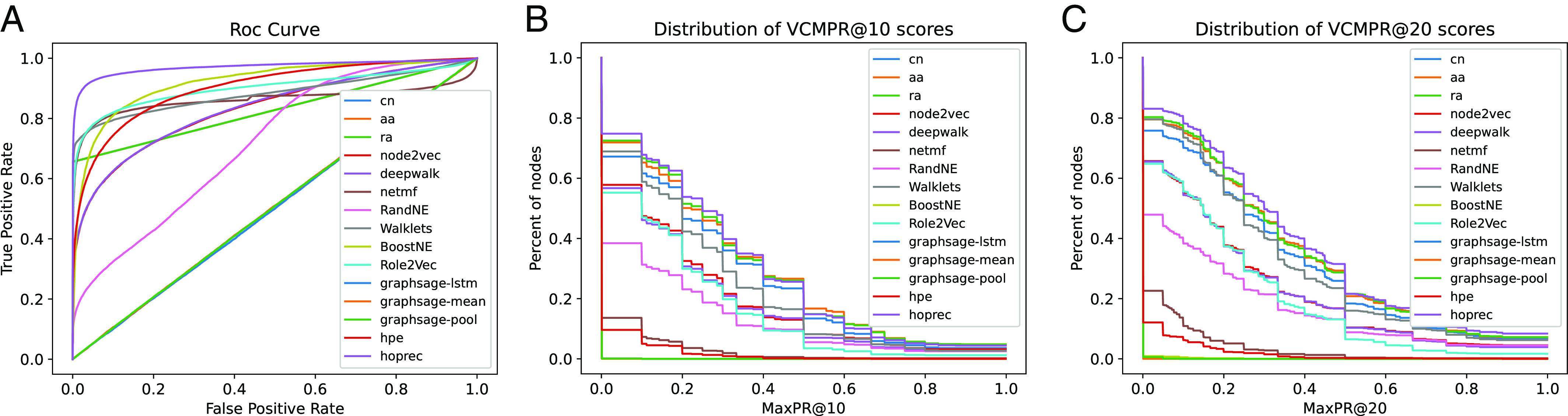
We train a number of important node embedding methods for transductive link prediction on the ogbl-collab dataset, a current link prediction benchmark on the Open Graph Benchmark Leaderboard (26). (*A*) We plot the standard ROC curves for each predictor. (*B*) We plot the distribution of the VCMPR@10 values. (*C*) We plot the distribution of VCMPR@20 values. The low VCMPR curves clearly show the weaknesses of existing methods. For all methods, at least 40% of the vertices have VCMPR@20 value of less than 0.3, which is quite low.

More significant are the VCMPR plots in Fig. 2, which pinpoint weaknesses in a way that single scores cannot capture. For example, the VCMPR@20 plot shows that, for all methods, at least 40% of the vertices have a VCMPR@20 score less than 0.4. Metrics like average scores or hit@k do not reveal such problems. (A choice of 20 is quite generous, since the average degree is 9.) One can perform a deeper investigation into this set to understand for which vertices the algorithm is failing. Many sparse applications of link prediction are often for personalization in recommendation systems, where a vertex-centric view is closer to the application (28, 29). Global metrics do not provide insights at a vertex level.

## Theoretical Explanation

We find a theoretical connection between the poor VCMPR and the low-dimensional aspect of embeddings. The standard scoring method for link prediction is the dot product (or Hadamard product) of the embedding vectors. We stress that almost all previous work follows this method (6–10, 18). We construct a framework that formalizes the notion of sparse ground truth that should be “detectable” by scores involving dot products of embedding vectors. Under this framework, we prove that the rank of the embedding vectors must be nearly linear. This is a strong lower bound that counters the general notion that low-dimensional embeddings with dot product–based scores are a good method for link prediction.

For link prediction, the ground truth is fundamentally sparse, since the number of edges is proportional is to the number of vertices. If n is the number of vertices, the total number of edges we expect to see is O(n). This is much smaller than n2, the total number of potential edges. Alternately, the “yes class” is a tiny fraction of the size of the “no class” of nonedges as observed by Lichtenwalter and Chawla (21).

We explain the theoretical setup. Through some training process, a node embedding method outputs a vector ${\vec{v}}_{i}\in R^{d}$ for each vertex i in the original graph. We represent these vectors by the d×n matrix V, where each column is an embedding vector. In the link prediction task, the final classifier/predictor uses the vectors ${\vec{v}}_{i}$ and ${\vec{v}}_{j}$ to determine whether (i,j) will become an edge. The most common score used for prediction is the dot product ${\vec{v}}_{i}\cdot {\vec{v}}_{j}$ or the cosine similarity ${\vec{v}}_{i}\cdot {\vec{v}}_{j}/\left(\right\|{\vec{v}}_{i}{\|}_{2}\|{\vec{v}}_{j}{\|}_{2})$^*^.

Given a vertex i, we wish to predict some of the edges incident to i. Our first step is to formalize what it means for the ground truth to be “sparse,” and for the signal to be “strong.”

### Sparse, Undirected Ground Truth.

The total number of edges in the undirected graph is linear in n, the number of vertices. So, we expect an average vertex i to be linked with a constant number of other vertices. Moreover, if j is a good prediction for i, then i should be a good prediction for j.

### Strong Signal in the Scores.

The set of scores with respect to i is the set $\left\{{\vec{v}}_{i}\cdot {\vec{v}}_{j}|j\neq i\right\}$. We want the true positives (the edges) to “stand out” among these scores. By the sparsity discussed above, we do not want more than a constant number of candidates to stand out. For the vertex i, let $D_{i}$ be the distribution over all other vertices, where the probability of j is $|{\vec{v}}_{i}\cdot {\vec{v}}_{j}|/\sum_{j\neq i}\left|{\vec{v}}_{i}\cdot {\vec{v}}_{j}\right|$. We can interpret the dot product as a relative likelihood that j is a potential neighbor of i.

For the signal to be strong, we expect the true positives to have significant probability mass in this distribution. Note that we may have false positives because of negative entries. (We take absolute values to ensure a distribution. Nonetheless, true positives should have high likelihood/probability in $D_{i}$.) This discussion motivates a key definition. Let ε>0 be a parameter; think of it as a small constant.

Definition 1. A pair of vertices (i,j) is called significant if: both the probability of j in $D_{i}$ and the probability of i in $D_{j}$ are at least ε.

This captures the notion that j is a good prediction for i and vice versa. Roughly speaking 1/ε samples from $D_{i}$ are enough to detect j. Moreover, for a given i, there are at most 1/ε vertices j that form significant pairs.

We formally prove that any set of vectors that contain a linear number of significant pairs must have near-linear rank.

Theorem 1. *Consider a set of vectors *${\vec{v}}_{1},{\vec{v}}_{2},\ldots ,{\vec{v}}_{n}\in R^{d}$ *that are polynomially bounded in length. (So for some constant* c, $\max_{i}\|{\vec{v}}_{i}{\|}_{2}/\min_{i}{\left\|{\vec{v}}_{i}\right\|}_{2}$$\leq n^{c}$*.) Suppose there are at least* δn *significant pairs among these vectors. Then,* $\mathrm{rank}\left(V\right)\geq \mathrm{poly}\left(\varepsilon ,\delta ,\log^{-1}n\right)\times n$.

The condition on polynomial boundedness is a technicality; for most applications, n is extremely large (millions or more), so a polynomial bound in vector length is quite reasonable. The theorem is proven in *Theoretical Details*.

Let us discuss the relevance of this theorem. The lower bound is quite strong with respect to the number of significant pairs. Even if (say) half the vertices participate in just one significant pair, the rank bound is still near-linear. By the contrapositive of Theorem 1, when the rank is small, then there exist o(n) significant pairs. This means that ground truth “signal” is drowned in the noise. Sampling according to the distributions $D_{i}$ will not generate enough edges, resulting in many nonedges predicted.

This behavior will lead to poor precision/recall at the individual vertex level, exactly as we see in practice. In *More on AUC and Local VCMPR-Type Measures*, we empirically see that the dot product scores between edges and nonedges are indistinguishable. In all our experiments, the sparse signal is hard to distinguish from the noise. On the other hand, an average edge has a high score than an average nonedge, resulting in high AUC scores. But this is not relevant in an actual prediction task, where we need to report the pairs that are most likely to be edges.

### The VCMPR Challenge.

Can we design low-dimensional node embeddings that get high VCMPR plots for link prediction?

Our theoretical results suggest some fundamental limitations, when using dot product based scores for prediction. Our empirical work shows that even generalizations like the Hadamard product do not give better results. Indeed, the overwhelming evidence is that a new idea is required. We believe that solution may need alternate geometries or kernels over low-dimensional vectors for the link prediction process. Recent work by Chanpuriya et al. (30) has suggested asymmetric factorizations and other methods to avoid weaknesses of low-dimensional embeddings. These techniques may help address this challenge.

We see our results as providing guidance for future work on graph representation learning. Theoretical frameworks that shed light on existing limitations direct us away for methods that might not hold promise. Our work underscores the importance for different kinds of measurements of performance for link prediction. We hope that the VCMPR challenge provides a concrete problem to tackle. Regardless, we strongly advocate the use of VCMPR@k measures for link prediction performance in sparse graph settings.

We note that there are link prediction settings where our analysis might not be applicable (more discussion in *Limitations*).

### More on AUC and Local VCMPR-Type Measures.

We give more insight into why AUC is a poor measure for link prediction. Recall that $\chi :V^{2}\to \left[0,1\right]$ denotes the predictor function for future edges, and $E_{\mathrm{test}}$ is the ground truth. For convenience, $\left(i,j\right)∼E_{\mathrm{test}}$ means that (i,j) is a uniform random sample from $E_{\mathrm{test}}$. The following lemma gives an alternative definition of the ROC-AUC.

Lemma 1 (section 7 of ref. 24). *The ROC-AUC of the predictor* χ *is*

Pr(i,j)∼Etest,(i′,j′)∼V2∖Etest[χ(i,j)>χ(i′,j′)].

In plain English, the AUC is the probability that an average ground truth edge has a higher score than an average nonedge. (Henceforth, nonedge refers to a pair not in $E_{\mathrm{test}}$.) But the ground truth set $E_{\mathrm{test}}$ has size O(|V|), because real-world graphs are sparse. The complement (true negatives) has size $\Omega (|V{|}^{2})$, which is many orders of magnitude larger than the ground truth. Therein lies the fundamental problem. If ground truth edges have above average score, the AUC will be high. It is possible that there are (say) $10|E_{\mathrm{test}}|$ nonedges with score higher than the ground truth edges, but the AUC is still 1−o(1). The sparsity of ground truth makes it possible to have large AUC with a weak predictor. Indeed, this is what seems to happen in all our experiments. We stress that the weaknesses of AUC were known before (20). Lichtenwalter and Chawla specifically point out issues for link prediction (21). Despite that, it is the default method for evaluations of link predictors based on low-dimensional embeddings.

#### VCMPR variants.

The main utility of VCMPR is that is a local measure, as opposed to a global measure such as Lemma 1. For link prediction, it is crucial to understand how a predictor behaves at a vertex level (how many of the top predictions for i are correct). The VCMPR was chosen for easy interpretability, and is a fairly permissive metric. If the ground truth degree of i is small, then the denominator for VCMPR is also small.

In general, one can go beyond VCMPR and compute other local metrics. For example, we can measure local NDCG (normalized discounted cumulative gain) at each vertex. Moreover, one can compute VCMPR using just the test edges or with both test and train edges, and define other variations of VCMPR@k. We discuss these variants in *SI Appendix* and perform extensive experimentation on our datasets. Across all these measures, we consistently see poor performance for link prediction. This is a strong indication that the embeddings are not capturing the structure of the graph.

## Broader Context and Related Work

There is a rich literature showing that low-dimensional embedding methods successfully perform the link prediction task (6–10, 18). Despite much empirical work on node embedding methods, there are fewer principled theoretical results on their behavior some recent papers address this topic (30–34). There is growing evidence that hand-tuned algorithms can outperform embedding methods for link prediction (11, 34). Recent work argues that certain low-dimensional embeddings cannot capture the cluster structure of real-world graphs (31).

The AUC is a fundamental metric used in ML and statistical applications. Fawcett has an excellent overview of AUC (24). Hand argues that AUC is deeply flawed because it creates a data-dependent reweighting of false positives vs. false negatives (20). Deeply relevant to our own work, Lichtenwalter and Chawla perform an excellent study AUC in the context of link prediction (21). They show that the sparsity of the ground truth is a hindrance for using AUC. They also suggest precision and recall measures, but not in the localized manner of the VCMPR plots. Unlike previous work on AUC and link prediction, our work specifically connects the low values of VCMPR measures to low-dimensional embeddings. Existing work shows weaknesses of sampled metrics (like AUC) in ranking a single list (27). The crucial difference is that VCMPR focuses on the ranking of many lists, one for each vertex.

The hits@k (and precision/recall@k) metrics are fundamental measures in many knowledge completion tasks (35). We note the significant difference from personalized link prediction in the application. For knowledge completion, the goal is to find the potential edges, regardless of the endpoints. In personalized settings, we are given a specific vertex and want to know the potential edges incident to that vertex. Hence, the VCMPR metric more directly captures the goal.

Graph representation learning is an immensely large topic. We mention the surveys (1, 2) and Chapter 23 in ref. 3. Our experiments use a large variety of contemporary and classic methods (4, 6–10, 18, 22, 23, 28, 36–39). We use the implementations of ref. 40. The embedding methods span many categories, based on random walks, random projections, matrix factorizations, shallow embeddings, as well as Graph Neural Nets. (More details are in *Experimental Results*.)

An increasing body of literature has attempted to build theoretical frameworks to study this wide array of embedding algorithms. Qi et al. (10) provide an overarching template of matrix factorization that subsumes many methods. Several results study the theoretical power of Graph Neural Networks (32, 33, 41). Closer to our work, some results have tried to understand the limitations (and power) of low-dimensional embeddings. We borrow many theoretical tools from a result showing the inability of low-dimensional Singular Value Decomposition to preserve the triangle structure of real-world networks (31). Chanpuriya et al give alternate geometric methods to circumvent these limitations (30). It is not clear how to use these asymmetric embeddings to perform (symmetric) link prediction for undirected graphs.

## Limitations

Our mathematical analysis focuses on settings that are globally sparse, yet locally dense. (The local density appears in terms of having a strong signal at a vertex centric level.) This setting is widespread in most social network settings, often measured as low global density and high clustering coefficients. But for settings like link prediction in protein–protein interaction (PPI) networks, clustering coefficients are low, and our vertex-centric analysis might not be meaningful. We run link prediction experiments on PPI networks and observe that AUC scores are themselves quite low. In many of these experiments, VCMPR scores might not add more value.

Another important setting for link prediction is knowledge discovery. In such settings, the overall density may itself be high (seen in drug interaction datasets), or the vertex-centric measure might not be relevant. In knowledge discovery, we may be trying to predict any new edge, not just edges that are incident to a specific (set of) vertices. For such problems, our theoretical analysis is not informative and VCMPR metrics might not be meaningful. We perform experiments on a dense drug interaction dataset, and observe that hits@k metrics are probably more informative.

We also note that the term “density” may have different meanings. We treat it in terms of the total number of edges divided by the total number of possible edges. The vertex-centric measure we define focuses on typical vertices which usually have a low degree.

Regardless, we believe that AUC has fundamental flaws and should not be used to measure link prediction performance.

## Theoretical Details

In this section, we prove Theorem 1. For the proof of our main theorem, we will use the following lemma stated first by Swanapoel (42).

Lemma 2 [Rank lemma]. *Consider any square matrix* $M\in R^{n\times n}$. *Then*

$$\mathrm{rank}\left(M\right)\geq \frac{|\sum_{i}M_{i,i}{|}^{2}}{\left(\sum_{i}\sum_{j}{\left|M_{i,j}\right|}^{2}\right)}.$$

We prove our main theorem.

Proof 1. (of Theorem 1) Let us first bin the vectors based on their length (the Euclidean norm $\|{\vec{v}}_{i}{\|}_{2}$). For integer i, let $B_{i}$ denote the set of vectors whose length is in $\left[2^{i},2^{i+1}\right)$. By the polynomial length in bound, there are most $c\log_{2}n$ bins of vectors, for some constant c.

Imagine labeling each vertex by the bin that its corresponding vector belongs to. We can label a pair (i,j) with the labels of i and j. Formally, the label of pair (i,j) is the label/bin pair $\left(B_{a},B_{b}\right)$ where ${\vec{v}}_{i}\in B_{a}$ and ${\vec{v}}_{j}\in B_{b}$. Observe that each vertex can have at most $c\log_{2}n$ labels; hence, the total number of pair labels is at most $c^{2}{\mathrm{lg}}^{2}n$. (We use lg to denote $\log_{2}$.) There are at least δn significant pairs. By averaging, there exists some pair label $\left(B_{a},B_{b}\right)$ (where a may be equal to b), such that there are at least $\delta n/c^{2}{\mathrm{lg}}^{2}n$ significant pairs with that label. In other words, for some values a,b, there are at least $\delta n/c^{2}{\mathrm{lg}}^{2}n$ significant pairs between $B_{a}$ and $B_{b}$. Call these the marked significant pairs. Let $B_{a}^{\prime }\subseteq B_{a}$ and $B_{b}^{\prime }\subseteq B_{b}$ be the set of vectors in these bins that participate in the significant pairs between $B_{a}$ and $B_{b}$.

By definition (Definition 1), each i can participate in at most 1/ε significant pairs. Hence, the number of marked significant pairs (which are all incident to $B_{a}^{\prime }$) is at most $|B_{a}^{\prime }|/\varepsilon$. As argued above, the number of marked significant pairs is at least $\delta n/c^{2}{\mathrm{lg}}^{2}n$. Hence, $|B_{a}^{\prime }|\geq \left(\varepsilon \delta /c^{2}{\mathrm{lg}}^{2}n\right)\times n$. (Similarly, for $|B_{b}^{\prime }|$.) For convenience, let C be $B_{a}^{\prime }\cup B_{b}^{\prime }$.

Let $V^{\prime }$ be the column matrix of the vectors in C. Let M be the Gram matrix ${\left(V^{\prime }\right)}^{T}\left(V^{\prime }\right)$. (For convenience, let us index M with respect to the original vertex labels. So $M_{\mathrm{ij}}={\vec{v}}_{i}\cdot {\vec{v}}_{j}$.). Observe that the rank of M is the rank of $V^{\prime }$, which is at most the original rank of V. By Lemma 2,

[1] $$\begin{array}{c} \mathrm{rank}\left(V\right)\geq \mathrm{rank}\left(V^{\prime }\right)=\mathrm{rank}\left(M\right)=\frac{|\sum_{i\in C}{\vec{v}}_{i}\cdot {\vec{v}}_{i}{|}^{2}}{\left(\sum_{i\in C}\sum_{k\in C}{\left|{\vec{v}}_{i}\cdot {\vec{v}}_{k}\right|}^{2}\right)}. \end{array}$$

Let us begin by lower bounding the numerator. Wlog, assume a≤b. By definition, C contains $B_{b}^{\prime }$. Moreover, $|B_{b}^{\prime }|\geq \left(\varepsilon \delta /c^{2}{\mathrm{lg}}^{2}n\right)\times n$. For each vector in $B_{b}^{\prime }\subseteq B_{b}$, the length is at least $2^{b}$. Note that diagonal entry ${\vec{v}}_{i}\cdot {\vec{v}}_{i}$ is precisely the squared length. Hence, for any $i\in B_{b}^{\prime }$, ${\vec{v}}_{i}\cdot {\vec{v}}_{i}\geq {\left(2^{b}\right)}^{2}$. Combining all our bounds,

[2] $$\begin{array}{c} \sum_{i\in C}\left|{\vec{v}}_{i}\cdot {\vec{v}}_{i}\right|\geq \left(\varepsilon \delta /c^{2}{\mathrm{lg}}^{2}n\right)\times n\times {\left(2^{b}\right)}^{2}=\left(\varepsilon \delta /c^{2}{\mathrm{lg}}^{2}n\right)\times 2^{2b}\times n. \end{array}$$

We now upper bound the denominator of Eq. **1**. Every vertex in C participates in a significant pair in C. Hence, for every i∈C, there exists a j∈C such that j has probability at least ε in the distribution $D_{i}$ (Definition 1). So,

$$\frac{|{\vec{v}}_{i}\cdot {\vec{v}}_{j}|}{\sum_{k\neq i}\left|{\vec{v}}_{i}\cdot {\vec{v}}_{k}\right|}\geq \varepsilon \;\;⟹|{\vec{v}}_{i}\cdot {\vec{v}}_{j}|\geq \varepsilon \sum_{k\in C}\left|{\vec{v}}_{i}\cdot {\vec{v}}_{k}\right|.$$

By squaring and applying the $l_{1}$, $l_{2}$-inequality,

$$|{\vec{v}}_{i}\cdot {\vec{v}}_{j}{|}^{2}\geq \varepsilon^{2}(\sum_{k\in C}|{\vec{v}}_{i}\cdot {\vec{v}}_{k}{\left|\right)}^{2}\geq \varepsilon^{2}\sum_{k\in C}{\left|{\vec{v}}_{i}\cdot {\vec{v}}_{k}\right|}^{2}.$$

By Cauchy–Schwartz, $|{\vec{v}}_{i}\cdot {\vec{v}}_{j}\left|\leq \right\|{\vec{v}}_{i}{|}_{2}{\left\|{\vec{v}}_{j}\right\|}_{2}$. All the vectors are in C, where the length is at most the maximum length in $B_{b}^{\prime }$. This bound is at most $2^{b+1}$. Hence, we deduce that

$${\left(2^{b+1}\right)}^{2}\geq \varepsilon^{2}\sum_{k\in C}|{\vec{v}}_{i}\cdot {\vec{v}}_{k}{|}^{2}⟹\sum_{k\in C}{\left|{\vec{v}}_{i}\cdot {\vec{v}}_{k}\right|}^{2}\leq 4\varepsilon^{-2}2^{2b}.$$

We can now upper bound the denominator of Eq. **1**. We have $\sum_{i\in C}\sum_{k\in C}{\left|{\vec{v}}_{i}\cdot {\vec{v}}_{k}\right|}^{2}\leq 4\varepsilon^{-2}2^{2b}n$. We combine this bound with the numerator bound of Eq. **2** and plug into Eq. **1**

[3] $$\text{rank}(V)\geq \frac{{\left[(\varepsilon \delta /c^{2}{\mathrm{lg}}^{2}n)\times 2^{2b}\times n\right]}^{2}}{4\varepsilon^{-2}2^{2b}n},$$

[4] $$\;\;=\frac{(\varepsilon^{4}\delta^{2}/c^{4}{\mathrm{lg}}^{4}n)\times 2^{2b}\times n^{2}}{4\times 2^{2b}\times n}.$$

We can cancel out $2^{2b}n$ to conclude that $\mathrm{rank}\left(V\right)\geq \left(\varepsilon^{4}\delta^{2}/4c^{4}{\mathrm{lg}}^{4}n\right)\times n$.

### On AUC and the Rank Bound.

As a final point, we note that the rank lower bound (and poor reliability performance) is consistent with high AUC scores. We give a simple construction demonstrating a low-dimensional embedding that gives nearly perfect AUC scores for an example sparse graph. This construction also highlights the primary weakness of AUC as a measure for link prediction.

Theorem 2. *There exists a connected bounded degree graph* G with n *vertices and a corresponding constant dimension embedding with the following properties. The AUC of edge prediction using the dot product score is* 1−o(1).

Proof 2. Consider a graph G first formed by a disjoint collection of triangles. To make it connected, we add an arbitrary spanning tree that connects all the triangles. We will construct a randomized embedding with a high probability of having AUC 1−o(1). By the probabilistic method, there must exist some embedding with such a large AUC.

For each triangle, let us assign an independent, Gaussian uniform random unit vector in $R^{d}$. All vertices in the triangle are assigned to this vector. Hence, for every edge (i,j), ${\vec{v}}_{i}\cdot {\vec{v}}_{j}=1$. Now, consider i and k that are not part of a triangle. Observe that ${\vec{v}}_{i}\cdot {\vec{v}}_{k}$ is distributed as the dot product of independent Gaussian random vectors. This is distribution as $N(0,1/\sqrt{d})$, a standard Gaussian with $1/\sqrt{d}$ SD. For sufficiently large constant c, the dot product is at most 1/4 with high probability. Since a 3/4-fraction of edges are in triangles, the average dot product of an edge is at least 3/4. For a uniform random pair, with high probability, the score is at most 1/4. Hence, the expected AUC is 1−o(1).

Note that in this construction there are $\Theta (n^{2})$ dot products with value $\Theta (1/\sqrt{d})$. The distributions $D_{i}$ will have a lot of noise, and the true signal (the dot product of 1) will have negligible mass.

## Experimental Results

We follow the standard setup for transductive link prediction experiments, as done in previous work. The experimental sections of the node2vec (7) or role2vec (18) papers provide good examples. The datasets are described in Table 3, taken from refs. 25 and 43. We also include two link prediction datasets from the Open Graph Benchmark (26), and two protein interaction datasets from refs. 44 and 45 We choose a dense dataset, ogbl-ddi, as a counterpoint to the other sparse datasets. In addition, we construct a small stochastic block model (SBM). We create an SBM with a block size of 50 and 200 such blocks. Within each block, an edge is inserted with probability 0.3. Pairs across blocks are connected with probability 0.3/n (where n is the number of vertices). So blocks are extremely dense, and there are very few edges across blocks.

**Table 3.** Dataset summary

	Nodes	Edges	mean degree	clust. coeff.	Trans- -itivity	Triangles
amazon	334K	925K	5	0.39	0.21	667K
dblp	317K	1.04M	6	0.63	0.31	2.24M
blogcatalog	10K	333K	64	0.46	0.09	5.61M
sbm	10K	75K	15	0.29	0.29	105K
ogbl-collab	235K	1.28M	9	0.71	0.34	3.66M
ogbl-ddi	4.27K	1.33M	625	0.63	0.57	27.3M
bioplex	11K	56.6K	10	0.10	0.06	29.3K
HI-II-14	4.3K	14K	6	0.05	0.03	6.62K

The edges of each dataset (graph) are split into a train and test set. We put 80% of the edges in the training set and 20% in the test set (called $E_{\mathrm{test}}$ earlier). We then apply an embedding method to embed each vertex into a 128-dimensional vector. We describe the embedding methods in the next subsection.

We also follow the usual setting for transductive link prediction when computing the ROC-AUC and PR-AUC. First, we randomly generate an equal number of negative edges to add to the test and train datasets. Now, we train a logistic classifier over the training dataset that computes a Hadamard product to predict edges. (In most cases, the optimal Hadamard product is just the dot product).

To compute the VCMPR plots, we sample 1,000 uniform random vertices. For each sampled vertex i, we sort all other vertices in decreasing order of the Hadamard product used by the classifier. We remove all pairs from the training data. We then compute the precision and recall of the top k entries of this sorted list, where the ground truth is the neighborhood of i in $E_{\mathrm{test}}$.

Plots for blogcatalog are given in Fig. 1 and Table 1. Results for ogbl-collab are in Fig. 2 and Table 2, and for the SBM are in Table 4. All other plots and results are given in *SI Appendix*.

**Table 4.** We run our experiments on a small

	ROC-AUC	PR-AUC	Avg VCMPR@10
Resource allocation	0.96	0.98	0.26
Adamic adar	0.96	0.98	0.26
Common neighbors	0.96	0.98	0.26
HOP-Rec	0.99	0.99	0.26
HPE	0.99	0.99	0.25
NetMF	0.99	0.99	0.26
GraRep	0.99	0.99	0.24
Walklets	0.99	0.99	0.25
Node2Vec	0.98	0.98	0.23
DeepWalk	0.98	0.98	0.24
BoostNE	0.98	0.98	0.16
RandNE	0.98	0.98	0.24
Role2Vec	0.97	0.97	0.21
GraphSage-MP	0.76	0.82	0.11
GraphSage-M	0.72	0.77	0.06
GraphSage-L	0.69	0.71	0.02

SBM Again we see the stark contrast between AUC and VCMPR scores. AUC scores are near perfect. But the VCMPR scores are quite low, under 0.25. See *SI Appendix*, Fig. S7.

### Embedding Methods.

We experiment on a large set of embedding methods, that subsume random walk methods, factorization methods, and deep models. When available, we use the implementations of these algorithms provided by the KarateClub library (40). We included HOP-Rec (37), a leading method for ogbl-collab on the OGB leaderboard (26). For completeness, we run some classic nonembedding methods, such as Common Neighbors (28), Adamic Adar (4) and Resource Allocation (36). These methods typically have good performance (within top 10) on the leaderboard datasets (26).

#### DeepWalk.

DeepWalk (6) is a classic shallow embedding model that uses uniform random walks.

#### Node2Vec.

Node2Vec (7) is another important shallow embedding model.

#### NetMF.

NetMF (10) is a matrix factorization–based model that approximates the DeepWalk matrix.

#### GraRep.

GraRep (39) is a direct matrix factorization–based model. We ran GraRep only on small graphs.

#### BoostNE.

BoostNE (23) is an ensemble matrix factorization–based embedding model.

#### RandNE.

RandNE (22) is a random projection–based embedding model.

#### Walklets.

Walklets (8) is a random walk–based embedding model that samples short random walks.

#### Role2Vec.

Role2Vec (18) is a random walk–based embedding model that embeds vertices based on attributed random walks.

#### GraphSage.

GraphSage (9) is an deep learning–based embedding algorithm. We use the unsupervised version of GraphSage, with the mean, LSTM, and max-pooling aggregators.

#### HPE.

HPE (38) is a random walk–based embedding model that embeds vertices based on preference edges.

#### HOP-Rec.

HOP-Rec (37) is an embedding model that combines random walks and matrix factorization.

### Key Observations.

#### High AUC values.

Across all datasets and a majority of methods, the ROC-AUC and PR-AUC values are quite high (more than 0.8). For brevity, we only plot the ROC curves. We can see the characteristic “away from diagonal” trend that leads to high AUC. The AUC values are given in Tables 1 and 4. Most methods give an AUC of at least 0.8 for all datasets; the maximum is at least 0.95. Consistently, the Walklets algorithm has strong performance in the AUC metric. The dblp dataset appears to be easier to learn, since all methods give high AUC scores.

#### Low VCMPR values.

Across all methods and all datasets, the VCMPR values are quite low. We compute these values for k=10,20,50 (that is, the top 10, 20, and 50 scores for each vertex). The low performance for k=10 is quite surprising, suggesting that the top scores are almost always non-edges. Note that VCMPR should go up for larger k, since recall will always increase. The average degrees in all the graphs are well below 50. So the VCMPR@50 is basically measuring recall, with a list that is much larger than the degree. Despite that, the values are low. For the amazon, dblp datasets, the average degree is at most 6, which is quite small. For that reason, we only measure VCMPR@10 and VCMPR@20. (For k=50, it would be measuring recall over a list of almost 10 times the size of ground truth list, which is not meaningful.)

For example, in the blog-Catalog dataset (Fig. 1 and Table 1), the highest average VCMPR values is 0.21. Most methods have an average VCMPR value of less than 0.1. This means that, on the average, among the top 10 scores for a vertex i, at most one of them is a ground truth edge. We see the noise “drowning” out the signal, just as the theory in *Theoritical Details* suggests. For the dblp and amazon datasets, we see the same phenomenon, though the numbers are somewhat higher. The AUC scores for amazon are remarkably high, above 0.8. But the average VCMPR values are mostly at 0.3. Note that the average degree is 6, since VCMPR@20 is measuring how many of those neighbors are within the top 20 scores. This is rather permissive from a prediction standpoint, and yet the values are quite low. Walklets performs somewhat better, but nowhere near what the AUC would suggest. (An average VCMPR@10 of 0.38 means that, for an average vertex, at most 38% of the top 10 scores are actually edges.) We see a similar story with the dblp dataset.

These results are evidence that the low-dimensional node embedding methods are missing much of the graph structure. The high AUC scores are misleading indicators of link prediction performance. In numerous cases, a predictor with an AUC of more than 0.8 has an average VCMPR of less than 0.05. We also compute the local NDCG and consider metrics with both test and train data. The poor performance is consistent across all these measures (detailed discussion in *SI Appendix*).

Quite surprisingly, we see this pattern even when evaluating non-embedding-based link prediction methods, such as the high-performing Resource Allocation method.

### SBM Experiments.

The AUC values are nearly perfect, and one might think that prediction is extremely accurate. But the average VCMPR@10 values are quite low, typically around 0.25. Again, the high AUC scores hide the fact that the link prediction is not accurate. We see again the sharp contrast between AUC and the actual predictive power.

### On Other Metrics.

We get analogous results for VCNDCG (vertex-centric normalized discounted cumulative gain). The VCNDCG plots are quite low, and average scores are lower than 0.2. This emphasizes that the vertex-centric view highlights the weaknesses of embedding-based link prediction. (Plots and more discussion in *SI Appendix*.)

We also experimented with the classic hits@k metric, which is popular for knowledge completion tasks (26, 35, 46). As discussed in the introduction, the hits@k is a global metric where a predicted score is compared against a sorted list of scores for ground truth edges and non-edges. We note that the parameter k is incomparable for hits@k and VCMPR@k, since the former considers a total list of edges/pair, while the latter considers a list of edges/pairs for each vertex. Table 2 and Fig. 2 highlight that hits@k can be quite large, while corresponding VCMPR scores can be low. We give more details for other datasets in *SI Appendix*.

### The Connection to Density.

The theory and experiments related the weakness of AUC for sparse link prediction problems, where the VCMPR is able to identify weak performance. As a counterpoint, we do experiments on the ogbl-ddi link prediction benchmark, which is a dense graph. The average degree is more than one-tenth of the number of vertices. For these data, the VCMPR numbers are higher and comparable to the AUC scores. More details are in *SI Appendix*.

### Results on PPI Networks, and the Clustering Coefficient Connection.

**Fig. 3.**
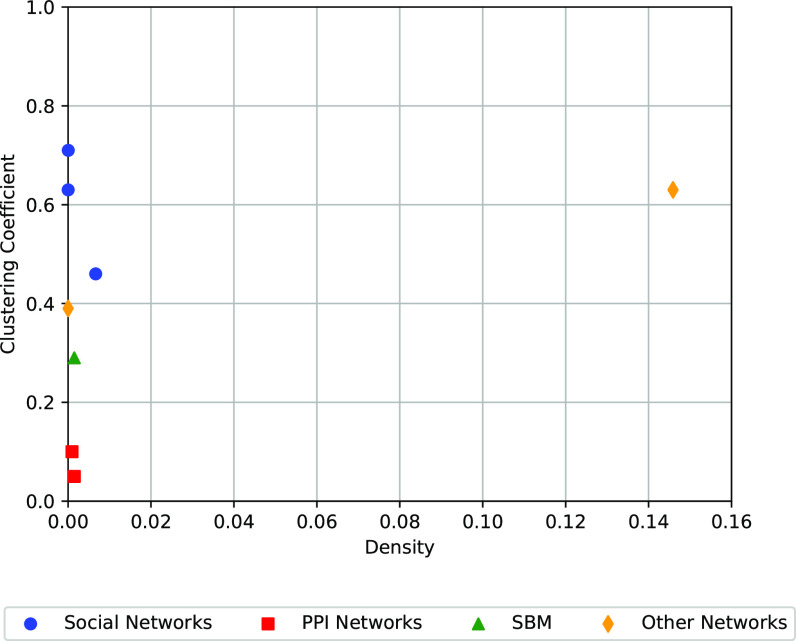
We plot the network density vs. clustering coefficient for all networks listed in Table 3. The social networks includes the blogcatalog, ogbl-collab, and dblp datasets. The PPI networks refers to bioplex and HI-II-14, while other networks refers to amazon, a product co-purchasing dataset and ogbl-ddi, a drug interaction dataset.

In Fig. 3, we plot the network density vs. clustering coefficient for all the datasets we experiment with. Observe that most of the social networks lie far above the PPI networks (which have low clustering). For the latter setting, our mathematical analysis is not as applicable. We perform the same collection of link prediction experiments on the PPI datasets. We observe that AUC scores tend to be lower (closer to 0.5). It is likely the AUC does not suffer from the same problem for other datasets. There are a few algorithms, like Walklets, HPE, and HOP-rec that have high AUC. Nonetheless, in all cases, the VCMPR scores are quite low, consistent with other datasets. More details are in *SI Appendix*.

## Exploration of Dot Products

In this section, we do a deeper investigation of the actual dot product values, to corroborate the theory in *Theoritical Details*. Theorem 1 implies that when the rank of the vectors is low, the sparse ground truth cannot have significantly higher dot products than the nonedges. As a case study, we focus on the blogCatalog dataset, though our results are consistent over other graphs.

Consider the set of vectors $\left\{{\vec{v}}_{i}\right\}$ output by some embedding method for blogCatalog. For each vertex i, we first compute the average dot product ${\vec{v}}_{i}\cdot {\vec{v}}_{j}$, for all edges (i,j) in the test set. Call this quantity $t_{i}$. Then, we compute the average dot product ${\vec{v}}_{i}\cdot {\vec{v}}_{j}$ for the top 50 nonedges (i,j). Call this quantity $f_{i}$. If we want to predictor to have detected the ground truth, we would want $t_{i}\gg f_{i}$.

**Fig. 4.**
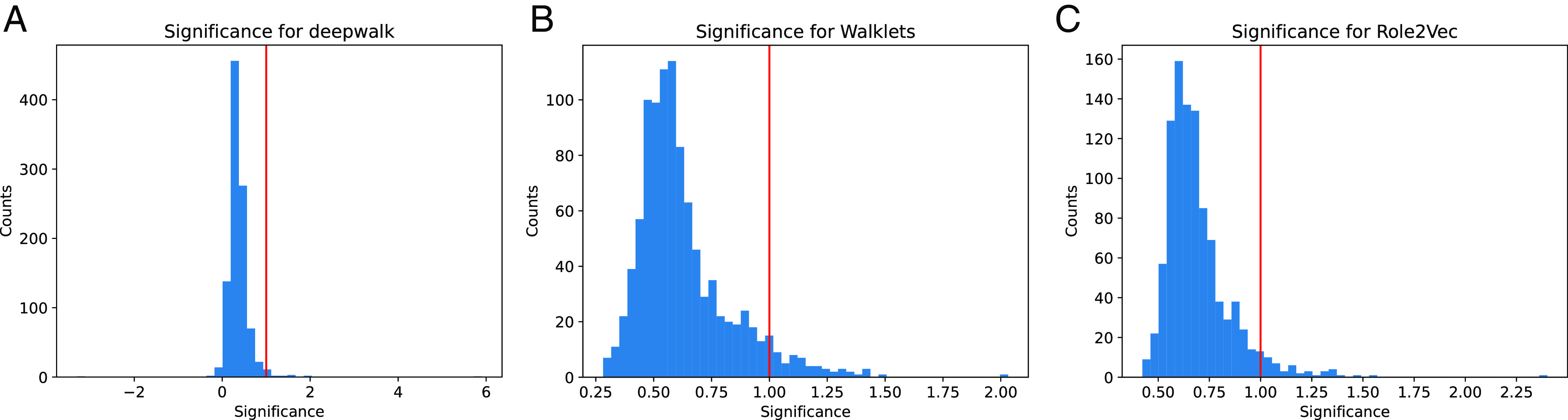
We plot the distributions of significance values for the embedding vectors generated by deepwalk in (*A*), Walkets in (*B*) and Role2Vec in (*C*), for the blog-Catalog dataset. Roughly speaking, the significance value for a vertex i is the average dot product with its neighbor vectors divided the average of the top 50 ${\vec{v}}_{i}\cdot {\vec{v}}_{j}$ values (varying over j). The plot gives the distribution (over vertices) of significance values. For ground truth to be captured, we would need the significance to be much higher than 1. Our theory predicts that this will not happen, and most significance values will be much smaller. This is exactly what we see in practice. The distribution of significance values is much lower than the red line, which is at 1.

So let us define the significance of i to be $t_{i}/f_{i}$. This is the ratio of the score of an average ground truth edge and the score of an average top-50 nonedge. The distributions (for a uniform random sample of 1,000 vertices) are given in Fig. 4 for the DeepWalk, Walklets, and Role2Vec embedding methods. We mark the significance of 1 as a red line. (For DeepWalk, there are some negative significance values, since the average dot product of test edges for some i is negative).

For a predictor that captures the ground truth, we would expect the distribution to have mass much to the right of the red line. As our theory predicts for the low-dimensional vectors, this is not the case. The distribution is to the left of the red line, so most vertices have a significance less than 1. This means that among the top 50 scores, nonedges dominate edges.

## Supplementary Material

Appendix 01 (PDF)

## Data Availability

Code used to produce all of the data have been deposited in https://github.com/nmenand/Link-Prediction-Experiments (47). The datasets used are publicly available and can be obtained from links in the corresponding citation. Blogcatalog3 can be obtained from the social computing data repository at ASU (22). Amazon and DBLP can be obtained from the Stanford network analysis project (43). ogbl-collab and ogbl-ddi can be obtained from the Open Graph Benchmark (26). HI-II-14 and Bioplex can be obtained from refs. 44 and 45.

## Footnotes

^*^In the latter case, we can just assume that the column vectors in V are normalized. So we will stick with the score being the dot product.
