# Supplementary material for: Link prediction using low-dimensional node embeddings: The measurement problem
Source: Proc Natl Acad Sci U S A. 2024 Feb 16;121(8):e2312527121. doi: 10.1073/pnas.2312527121 (PMC10895345; doi:10.1073/pnas.2312527121)
Supplement: Supplementary file 1 — Appendix 01 (PDF) [file pnas.2312527121.sapp.pdf]

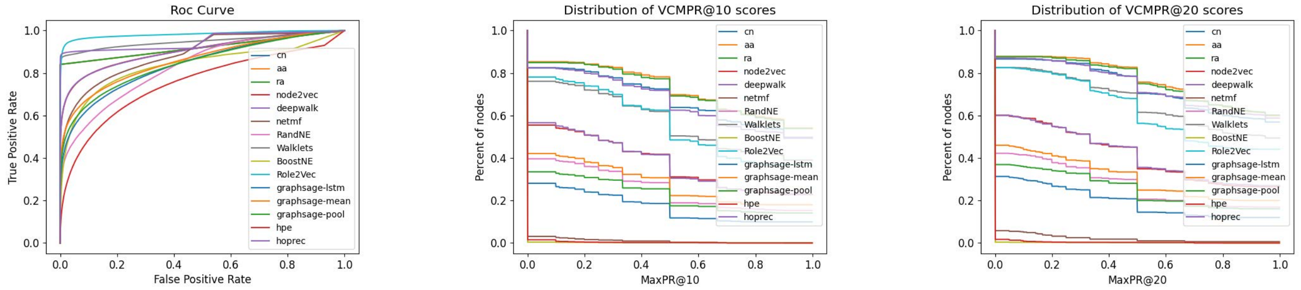

(a) ROC curve **(b)** VCMPR@10 **(c)** VCMPR@20

Fig. 5. We show results for `dblp` dataset (43). The left figure has the standard ROC curves for each predictor. In the middle and right figures, we plot the distribution of VCMPR@k values, for $k = 10, 20$. The `dblp` dataset has a small degree of 6, which is smaller than the choice of k. The ROC curves are quite high, consistent with the literature. But the VCMPR values show that the predictor performance is not strong. The data is summarized in Tab. 5.

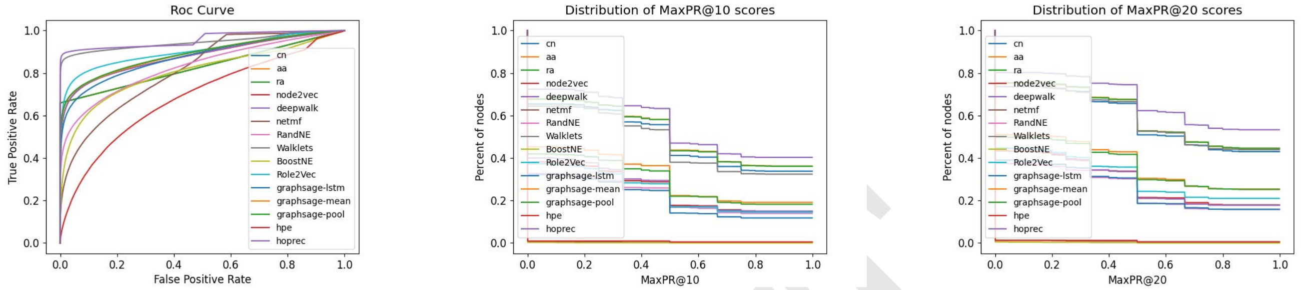

(a) ROC curve **(b)** VCMPR@10 **(c)** VCMPR@20

Fig. 6. We show results for `amazon` dataset (43). The `amazon` dataset also has a small average degree of 5, so $k = 20$ is significantly large for this dataset. The AUC scores are high, more than 0.8 for almost all cases. The VCMPR values are quite low. Even for $k = 20$, the plots are quite low. There are very few vertices with VCMPR values above 0.7. The data is summarized in Tab. 6.

DRAFT

Supporting Information for "Link prediction using low-dimensional node embeddings: the measurement problem"

A. Details on experiments

We give details on all the embedding methods used in our experiments. We have included HOP-REC, an embedding method that performs highly on the Open Graph Benchmark link prediction leaderboards (26). We explore general node embedding models, such as Node2Vec (7), NetMF (10), Role2Vec (18), as well as node embedding models specific designed for recommendations, such as Heterogenous Preference Embedding (HPE) (38) and HOP-Rec (37). Also, for the sake of completeness, we show results on some classic non-embedding methods, such as Common Neighbors (28), Adamic Adar (4), and Resource Allocation (36).

DeepWalk: DeepWalk (6) is a classic shallow embedding model that uses uniform random walks paired with a skip-gram language model to learn a low dimensional embedding. DeepWalk aims to embed nodes with similar neighborhoods (nodes with high second order proximity) close together. We ran DeepWalk with the following parameters, dimension $d = 128$, window size $w = 5$, walks per vertex $\gamma = 10$, walk length $t = 80$.

Node2Vec: Node2Vec (7) is another important shallow embedding model that expands on the random walks used in DeepWalk by introducing a transition probability associated with the 2nd order random walks. These two parameters correspond to the random walk behaving more like BFS or DFS, and is meant to help balance between the walk staying near the start vertex and exploring the graph. We ran Node2Vec with the following parameters, dimension $d = 128$, window size $w = 5$, walks per vertex $\gamma = 10$, walk length $t = 80$, $p = 1$, $q = 1$.

NetMF: NetMF (10) is a matrix factorization based model that approximates the DeepWalk matrix for a graph. The NetMF paper also showed that the previous random walk based models can be expressed in terms of matrix factorization with closed forms. We ran NetMF with the following parameters, dimension $d = 128$, 10 SVD iterations, and 2 PMI matrix powers.

BoostNE: BoostNE (24) is an ensemble matrix factorization based embedding model. It iteratively factorizes the residual of the connectivity matrix found by NetMF to produce multiple weak embedding representations. These are combined with a gradient boosting technique to produce a final embedding. We ran BoostNE with the following parameters $d = 128$, 16 boosting iterations, and for the NetMF calls, we use 2 PMI matrix powers.

RandNE: RandNE (23) is a random projection based embedding model that embeds the graph by using a Gaussian random projection. We ran RandNE with dimension $d = 128$, iterations $q = 2$ and an unweighted average $\alpha_0 = \alpha_1 = 0.5$.

Walklets: Walklets (8) is a random walk based embedding model that generates multi-scale relationships of vertices by sub sampling short random walks on the vertices of the graph. The parameters we choose for Walklets are dimension $d = 128$, window size $w = 4$, walks per vertex $\gamma = 10$, walk length $t = 80$.

Role2Vec: Role2Vec (18) is a random walk based embedding model that embeds vertices based on attributed random walks. An attributed random walk is a random walk on adjacent vertex types, where a type is defined by Weisfeiler-Lehman structural features. We set the parameters of Role2Vec to be $d = 128$, window size $w = 2$, walks per vertex $\gamma = 10$, walk length $t = 80$.

GraphSage: GraphSage (9) is an deep learning based embedding algorithm. We use the unsupervised version of GraphSage, with the mean, LSTM and max-pooling aggregators. We set the parameters of GraphSage to have output dimension $d = 128$, number of iterations 10,000, and the identity dimension to be 128.

HPE: HPE (38) is an embedding method specifically designed for recommender systems. It first constructs a preference matrix, then creates an embedding of this preference matrix using random walks. We set the parameters of HPE to be dimension to be 128, number of negative samples per positive to be 5, window size 5, and learning rate 0.025.

HOP-REC: HOP-REC (37) is a embedding method designed specifically for recommendations. It combines factorizations of the adjacency matrix and higher order matrices approximated by random walks. We set the parameters of HOP-REC to be dimension to be 128, number of updates to be 500, number of negative samples per positive to be 5, window size 5, and learning rate 0.025.

A. Results on different datasets. We analyze the above models on the following datasets. The `amazon` product co-purchasing network represents products that are frequently purchased together as edges between vertices in the graph (43). The `dblp` collaboration network is an unweighted graph where edges represents two authors publishing 1 or more papers together (43). The `blog-Catalog` social network represents bloggers and friendships between them (22). The `ogbl-ddi` network represents interactions between different drugs (26). The `ogbl-collab` network represents authors and collaborations between them (26). These graphs vary in size, average degree,

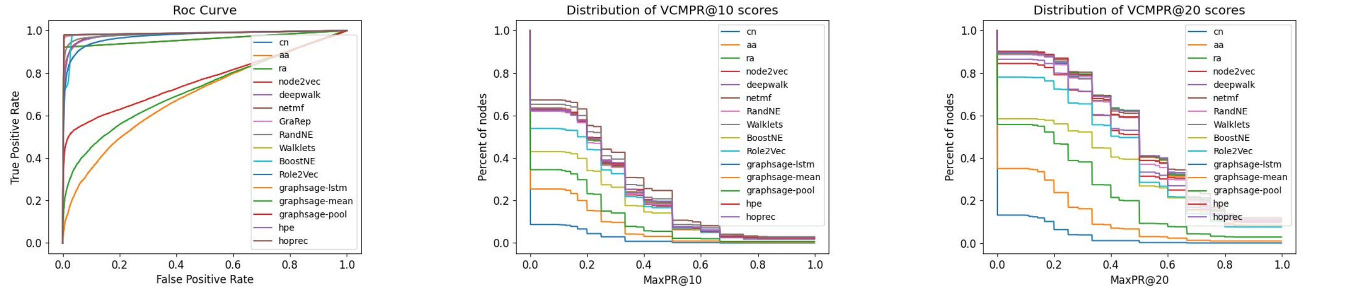

(a) ROC Curve **(b)** VCMPR@10 **(c)** VCMPR@deg20

Fig. 7. We show results for a small SBM. The SBM has a block size of 50 and 200 blocks. The AUC scores are exceptional, over 0.97 for almost all cases. However the VCMPR values are quite low. The VCMPR@10 scores are quite low, under 0.3. Even for $k = 20$, there are no methods that score above 0.5, despite the fact that at this threshold is more than half of the size of the block with the training edges removed. The data is summarized in Tab. 4.

	ROC AUC	PR AUC	Avg VCMPR @10	Avg VCMPR @20
Common Neighbors	0.92	0.96	0.65	0.71
Adamic Adar	0.92	0.96	0.70	0.75
Resource Allocation	0.92	0.96	0.70	0.74
Role2Vec	0.98	0.99	0.52	0.60
Walklets	0.95	0.97	0.54	0.63
HOP-Rec	0.95	0.97	0.64	0.71
Deepwalk	0.90	0.93	0.37	0.41
Node2Vec	0.90	0.93	0.40	0.43
NetMF	0.89	0.91	0.02	0.03
GraphSage-M	0.86	0.89	0.27	0.30
GraphSage-MP	0.84	0.88	0.23	0.26
GraphSage-L	0.84	0.87	0.17	0.20
BoostNE	0.83	0.87	0.00	0.00
RandNE	0.83	0.85	0.26	0.28
HPE	0.75	0.79	0.00	0.01

Table 5. This table complements Fig. 5, which has results from link prediction on the `dblp` dataset. We give the ROC-AUC, PR-AUC, and average VCMPR@k for $k = 10, 20$. The AUC numbers are extremely large for real data, close to 0.9, with all methods showing good scores. Comparatively, the average VCMPR@10 numbers are low. `Walklets` gets a score of 0.56, but other methods are below 0.4.

	ROC AUC	PR AUC	Avg VCMPR @10	Avg VCMPR @20
Adamic Adar	0.83	0.91	0.51	0.60
Resource Allocation	0.83	0.91	0.52	0.60
Common Neighbors	0.82	0.91	0.49	0.58
HOP-Rec	0.96	0.97	0.56	0.67
Walklets	0.95	0.96	0.47	0.59
Role2Vec	0.90	0.93	0.24	0.31
Deepwalk	0.88	0.91	0.26	0.29
Node2Vec	0.88	0.91	0.25	0.29
GraphSage-M	0.88	0.92	0.31	0.37
GraphSage-MP	0.88	0.92	0.29	0.37
GraphSage-L	0.87	0.90	0.21	0.26
RandNE	0.83	0.87	0.22	0.26
NetMF	0.81	0.82	0.00	0.01
BoostNE	0.80	0.85	0.00	0.00
HPE	0.69	0.71	0.01	0.01

Table 6. This table complements Fig. 6, which has results from link prediction on the `amazon` dataset. We give the ROC-AUC, PR-AUC, and average VCMPR@k for $k = 10, 20$. The AUC numbers are quite high, with a highest of 0.97. But the average VCMPR number is quite low. Even the highest for `Walklets` is at 0.58, while other methods are lower than 0.4. The average degree is `amazon` is 5, so a choice of $k = 20$ is quite large.

DRAFT

and clustering coefficient as shown in Tab. 3. We train each model on 90% of the edges of the graph and withhold 10% for testing. The results for `amazon` can be found in Fig. 6 and Tab. 6, for `dblp` in Fig. 5 and Tab. 5, for `blog-Catalog` in Fig. 1 and Tab. 1, for `ogbl-collab` in Fig. 2 and Tab. 2, and for `ogbl-ddi` in Fig. 8 and Tab. 7.

We do some small scale experiments with simple Stochastic Block Models (SBMs) to make our point more compelling. We create an SBM with a block size of 50 and 200 such blocks. Within each block, an edge is inserted with probability 0.3. Pairs across blocks are connected with probability $0.3/n$ (where n is the number of vertices). So blocks are extremely dense, and there are few edges across blocks. We show the results in Tab. 4 and Fig. 7.

B. The connection to graph density. We show results on the dense `ogbl-ddi` dataset. In Tab. 7, we can see that VCMPR scores are quite high, and in many cases, almost the same as AUC scores. For the leading `HOP-rec` and `Walklets` algorithms, the scores are quite close to each other. This is an empirical converse of our main result theory that connects poor link prediction performance for low-dimensional embeddings to sparsity of the ground truth data. When the ground truth is dense, then both AUC and VCMPR suggest that the algorithms are performing well in link prediction.

C. Results on PPI datasets. Unlike other datasets, PPI networks tend to have low clustering coefficients. Hence, they are not covered by our theoretical analysis. We perform the same link prediction experiments on the PPI datasets, `Bioplex` (45) and `HI-II-14` (44), described by Fig. 9 and Fig. 10. We observe that AUC scores are already quite low, typically between 0.6 and 0.8. There are few notable examples like *Walklets* and *HOP-rec* on the `Bioplex` where AUC scores are above 0.85, despite having VCMPR@10 scores below 0.2 and VCMPR@50 scores below 0.35. Observe that *NetMF* has higher VCMPR scores than *Walklets*, despite having lower ROC-AUC and PR-AUC scores. But in all cases, the VCMPR scores are extremely low. Overall, AUC is typically sufficient to demonstrate poor performance of link prediction algorithms for such datasets. Hence, VCMPR may have limited utility for such settings.

D. Exploration of Normalized Discounted Cumulative Gain. We also compute a vertex-centric Normalized Discounted Continuous Gain (NDCG). NDCG is a metric that analyzes how well a ranking method ranks relevant documents (47). Similarly to VCMPR, we compute VCNDCG@k for some threshold k. Formally, VCNDCG@k is computed as follows. For a given vertex i of non-zero degree, we rank all other vertices j in decreasing order of their scores. Let L be a list of the binary ground truth values ordered by their ranking. Let I, the ideal ranking, be a list of binary ground truth values in sorted order. We consider all ground-truth lists only contain values of 0 or 1, i.e. relevant or not. Then VCNDCG@k for the vertex i is defined as

$$\text{VCDCG@k} = \sum_{s=1}^{k} \frac{L_s}{\log_2(s+1)} \qquad \text{VCIDCG@k} = \sum_{s=1}^{k} \frac{I_s}{\log_2(s+1)}$$

$$\text{VCNDCG@k} = \frac{VCDCG@k}{VCIDCG@k}$$

We compute VCNDCG for all data-sets and plot their scores in Fig. 12. Just as with VCMPR, the scores are quite low, which indicates that the low-dimensional embeddings have poor overall performance.

E. Comparison with Hits@k. As mentioned in the main body, the hits@k is a global metric appropriate for knowledge completion tasks. The natural

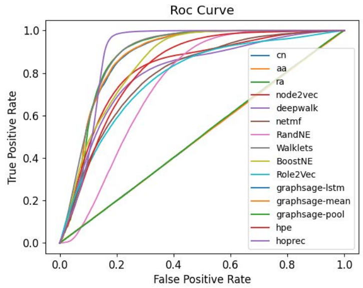

(a) `ogbl-ddi` ROC-AUC

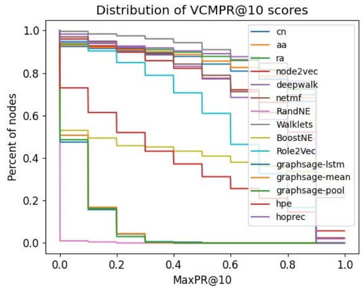

(b) `ogbl-ddi` VCMPR@10

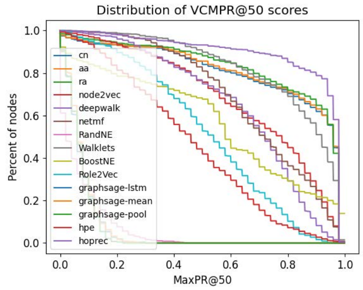

(c) `ogbl-ddi` VCMPR@50

Fig. 8. We compute VCMPR curves for the `ogbl-ddi` dataset.

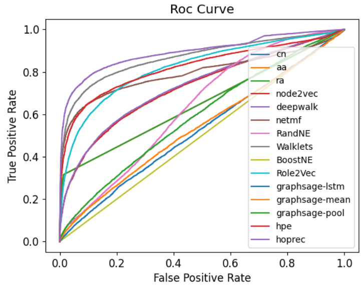

(a) `bioplex` ROC-AUC

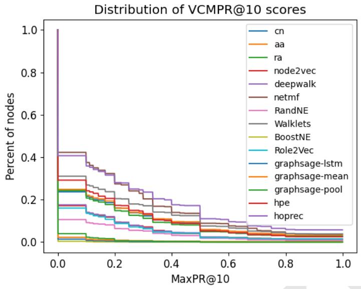

(b) `bioplex` VCMPR@10

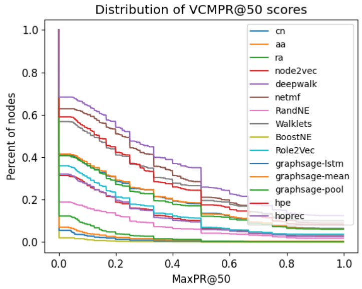

(c) `bioplex` VCMPR@50

Fig. 9. We compute VCMPR curves for the `bioplex` dataset.

DRAFT

	ROC-AUC	PR-AUC	Avg VCMPR @10	Avg VCMPR @50
Common Neighbors	0.88	0.80	0.77	0.82
Adamic Adar	0.88	0.80	0.78	0.82
Resource Allocation	0.88	0.81	0.80	0.84
Deepwalk	0.80	0.75	0.71	0.66
Node2Vec	0.82	0.78	0.72	0.68
NetMF	0.74	0.74	0.74	0.70
RandNE	0.75	0.63	0.00	0.06
Walklets	0.89	0.83	0.84	0.83
BoostNE	0.85	0.79	0.41	0.57
Role2Vec	0.78	0.74	0.59	0.52
GraphSage-M	0.50	0.50	0.08	0.08
GraphSage-MP	0.50	0.50	0.07	0.07
GraphSage-L	0.50	0.50	0.07	0.07
HPE	0.82	0.76	0.38	0.46
HOP-rec	0.89	0.81	0.82	0.90

Table 7. This table complements Fig. 8, which has results from link prediction on the `ogbl-ddi` dataset. We give the ROC-AUC, PR-AUC average VCMPR@k for $k = 10, 50$.

	ROC-AUC	PR-AUC	Avg VCMPR @10	Avg VCMPR @50
Common Neighbors	0.65	0.81	0.10	0.19
Adamic Adar	0.65	0.81	0.10	0.19
Resource Allocation	0.65	0.81	0.10	0.18
Deepwalk	0.73	0.76	0.06	0.12
Node2Vec	0.73	0.76	0.06	0.11
NetMF	0.80	0.85	0.16	0.31
RandNE	0.63	0.61	0.04	0.07
Walklets	0.86	0.90	0.13	0.27
BoostNE	0.50	0.75	0.00	0.00
Role2Vec	0.82	0.84	0.05	0.08
GraphSage-M	0.54	0.55	0.01	0.02
GraphSage-MP	0.59	0.59	0.01	0.02
GraphSage-L	0.53	0.53	0.00	0.01
HPE	0.82	0.86	0.11	0.26
HOP-rec	0.89	0.92	0.18	0.34

Table 8. This table complements Fig. 9, which has results from link prediction on the `Bioplex` dataset. We give the ROC-AUC, PR-AUC, average VCMPR@k for $k = 10, 50$.

choice of k for VCMPR is the average degree (or maybe twice average degree). For the hits metric, the k can vary depending on the instance. On the OGB leaderboard, common choices are $k = 20, 50$. We performed a comparison of all methods on the `ogbl-collab` dataset, where we see that VCMPR scores are much lower than then hits scores.

We also perform the same experiments on the `sbm` datasets we generated. We simply set the k parameter to 20, for both VCMPR and hits. We see that again, hits scores are significantly higher than VCMPR scores. The results are summarized in Tab. 11. For example, the HOP-rec method has a hits@20 value of 0.83, but the average VCMPR@20 is 0.49. There are numerous hits values above 0.65, where the average VCMPR@20 is less than 0.5.

For the dense `ogbl-ddi` dataset, the recommended metric on OGBL is hits@20. Here, we see the opposite: VCMPR scores are large, but the hits scores are low. This is another indication that the hits@k and VCMPR metrics are fundamentally different.

We also perform the same experiment on a PPi dataset, specifically the `Bioplex` dataset, with the number of negative instances set to 10% of the number of edges, consistent with the numbers chosen in the OGB datasets. We compare VCMPR and hits with k set to 50. Here we see that VCMPR scores are again lower than then hits scores, for example Role2Vec which has a VCMPR@50 of 0.08 but a hits@50 of 0.47.

F. Results on entire datasets. For the same setup as described in the paper, we compute VCMPR@k plots for the entire dataset, not just the graph consisting of E_{test}. This includes edges seen in training. This allows us to closely examine the local structure of the embedding. Under this setting, VCMPR@k is defined as follows.

$$\text{VCMPR@k for vertex } i = \frac{t_i(k)}{\min(k, D_i)}$$

where D_i is the degree of vertex i in the entire graph $G = (V, E)$. We report the scores in Fig. 13 and Fig. 14. As expected the scores are much higher. However, we see that in general, across all data-sets, thresholds, and methods, very few vertices have a VCMPR of 0.8, despite the embedding having seen 80% of the graph's edges. As before VCMPR curves drop quite steeply. This shows that the low-dimensional node embedding methods are not capturing much of the graph structure.

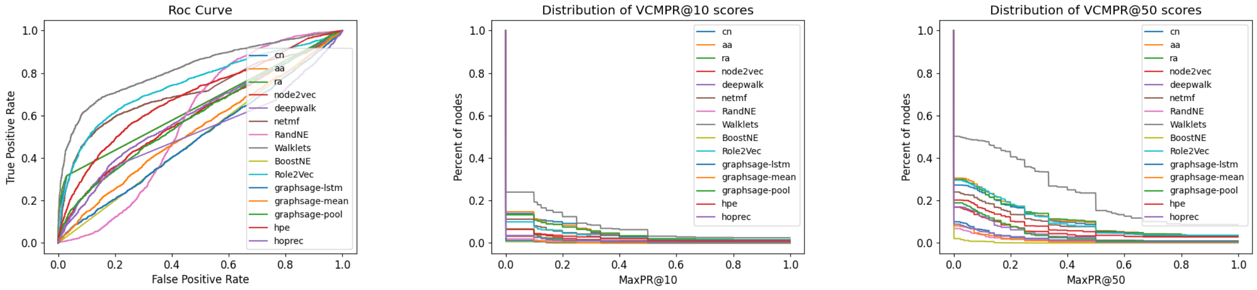

(a) `HI-II-14` ROC-AUC **(b)** `HI-II-14` VCMPR@10 **(c)** `HI-II-14` VCMPR@50

Fig. 10. We compute VCMPR curves for the `HI-II-14` dataset.

	ROC-AUC	PR-AUC	Avg VCMPR @10	Avg VCMPR @50
Common Neighbors	0.64	0.79	0.05	0.10
Adamic Adar	0.65	0.79	0.05	0.11
Resource Allocation	0.65	0.79	0.05	0.11
Deepwalk	0.61	0.62	0.02	0.04
Node2Vec	0.60	0.62	0.02	0.04
NetMF	0.71	0.75	0.03	0.09
RandNE	0.58	0.50	0.01	0.01
Walklets	0.82	0.85	0.08	0.24
BoostNE	0.50	0.75	0.00	0.00
Role2Vec	0.75	0.77	0.03	0.05
GraphSage-M	0.53	0.54	0.00	0.01
GraphSage-MP	0.60	0.62	0.01	0.04
GraphSage-L	0.50	0.51	0.00	0.02
HPE	0.69	0.70	0.02	0.07
HOP-rec	0.53	0.58	0.01	0.02

Table 9. This table complements Fig. 10, which has results from link prediction on the `HI-II-14` dataset. We give the ROC-AUC, PR-AUC, average VCMPR@k for $k = 10, 50$.

G. Results using a variable threshold for VCMPR. We also investigate the use of a variable threshold in our evaluation. We compute VCMPR as follows. Let D_i be the degree of vertex i in the original graph $G = (V, E)$ and d_i be the degree of i in the test graph $G_{test} = (V, E_{test})$. Then VCMPR@Deg(v) is defined as follows:

$$\text{VCMPR@Deg(i) for vertex } i = \frac{t_i(D_i)}{\min(D_i, d_i)}$$

We set D_i and d_i to not be the same to ensure each threshold is sufficiently large. This experiment is motivated by the fact that in some graphs, the degree of vertices tends to obey a power law distribution. Under such a distribution, a small portion of vertices are incident to a large portion of the edges. Thus, variable thresholds may be more appropriate. The results of these experiments are in Fig. 11 and Tab. 12, where we see low scores as before. The performance of all methods is extremely low in comparison to the AUC scores.

DRAFT

	blogcatalog	amazon	dblp	sbm	collab	ddi	bioplex	HI-II-14
	Avg VCNDCG @50	Avg VCNDCG @10	Avg VCNDCG @10	Avg VCNDCG @10	Avg VCNDCG @10	Avg VCNDCG @50	Avg VCNDCG @10	Avg VCNDCG @10
Common Neighbors	0.23	0.30	0.49	0.15	0.21	0.82	0.07	0.03
Adamic Adar	0.24	0.31	0.53	0.15	0.23	0.82	0.07	0.03
Resource Allocation	0.24	0.31	0.2	0.15	0.23	0.84	0.06	0.03
Deepwalk	0.04	0.15	0.26	0.14	0.14	0.66	0.04	0.01
Node2Vec	0.03	0.15	0.27	0.14	0.14	0.68	0.04	0.00
NetMF	0.10	0.00	0.03	0.18	0.02	0.70	0.13	0.03
RandNE	0.00	0.13	0.18	0.14	0.09	0.07	0.03	0.00
Walklets	0.14	0.34	0.42	0.17	0.20	0.05	0.09	0.05
BoostNE	0.00	0.00	0.00	0.12	0.00	0.53	0.00	0.00
Role2Vec	0.02	0.17	0.43	0.15	0.15	0.52	0.04	0.02
GraphSage-M	0.01	0.18	0.17	0.04	0.00	0.08	0.00	0.00
GraphSage-MP	0.02	0.17	0.15	0.07	0.00	0.07	0.01	0.01
GraphSage-L	0.01	0.12	0.11	0.01	0.00	0.07	0.00	0.00
HPE	0.21	0.00	0.00	0.17	0.02	0.44	0.07	0.01
HOP-rec	0.26	0.39	0.50	0.15	0.25	0.85	0.12	0.01

Table 10. This table complements Fig. 12, which plots the Normalized Discounted Cumulative Gain of each method over multiple datasets. We give the average NDCG. We see that across all datasets, the scores are very low, typically below 0.2. Since `blog-Catalog` and `ogbl-ddi` have high datasets, we set the parameter to be 50.

Comparison of VCMPR to Hits

	sbm		collab		ddi		bioplex	
	VCMPR @20	Hits @20	VCMPR @20	Hits @50	VCMPR @50	Hits @20	VCMPR @50	Hits @50
Common Neighbors	0.50	0.52	0.31	0.53	0.82	0.18	0.19	0.32
Adamic Adar	0.50	0.74	0.34	0.65	0.82	0.18	0.19	0.32
Resource Allocation	0.50	0.74	0.33	0.65	0.84	0.05	0.18	0.32
Deepwalk	0.44	0.53	0.21	0.22	0.66	0.01	0.12	0.31
Node2Vec	0.44	0.59	0.21	0.22	0.68	0.02	0.11	0.28
NetMF	0.50	0.73	0.03	0.35	0.70	0.00	0.31	0.58
RandNE	0.50	0.67	0.15	0.09	0.06	0.05	0.07	0.09
Walklets	0.49	0.75	0.29	0.59	0.83	0.02	0.27	0.64
BoostNE	0.31	0.64	0.00	0.08	0.57	0.04	0.00	0.00
Role2Vec	0.42	0.52	0.21	0.44	0.52	0.00	0.08	0.47
GraphSage-M	0.10	0.11	0.00	0.00	0.08	0.00	0.02	0.09
GraphSage-MP	0.23	0.44	0.00	0.00	0.07	0.00	0.02	0.10
GraphSage-L	0.03	0.04	0.00	0.00	0.07	0.00	0.01	0.07
HPE	0.50	0.69	0.02	0.21	0.46	0.00	0.26	0.54
HOP-rec	0.49	0.83	0.35	0.66	0.90	0.00	0.34	0.71

Table 11. This table compares VCMPR to Hits of each method over multiple data-sets.

DRAFT

VCMPR@Deg(V)

	blog-catalog	amazon	dblp	sbm	collab	ddi	bioplex	HI-II-14
Common Neighbors	0.16	0.14	0.35	0.08	0.17	0.65	0.05	0.02
Adamic Adar	0.16	0.15	0.38	0.08	0.18	0.66	0.05	0.02
Resource Allocation	0.16	0.14	0.38	0.08	0.19	0.69	0.05	0.02
Deepwalk	0.03	0.07	0.18	0.08	0.12	0.45	0.03	0.01
Node2Vec	0.02	0.07	0.18	0.08	0.11	0.49	0.03	0.01
NetMF	0.06	0.00	0.01	0.10	0.02	0.46	0.10	0.02
RandNE	0.00	0.06	0.12	0.08	0.06	0.30	0.02	0.00
Walklets	0.09	0.19	0.29	0.10	0.15	0.63	0.06	0.04
BoostNE	0.00	0.00	0.00	0.07	0.00	0.49	0.00	0.00
Role2Vec	0.01	0.11	0.31	0.08	0.11	0.35	0.02	0.01
GraphSage-M	0.01	0.08	0.09	0.03	0.00	0.07	0.00	0.00
GraphSage-MP	0.01	0.07	0.09	0.03	0.00	0.07	0.00	0.01
GraphSage-L	0.01	0.05	0.06	0.01	0.00	0.07	0.00	0.00
HPE	0.12	0.00	0.00	0.09	0.01	0.40	0.05	0.01
HOP-rec	0.18	0.22	0.36	0.08	0.19	0.40	0.09	0.01

Table 12. This table complements Fig. 11, which plots the VCMPR scores using a variable threshold based on the deg(v). We give the average scores here. For `amazon` and `dblp`, we see a slight drop in scores when compared to the fixed thresholds.

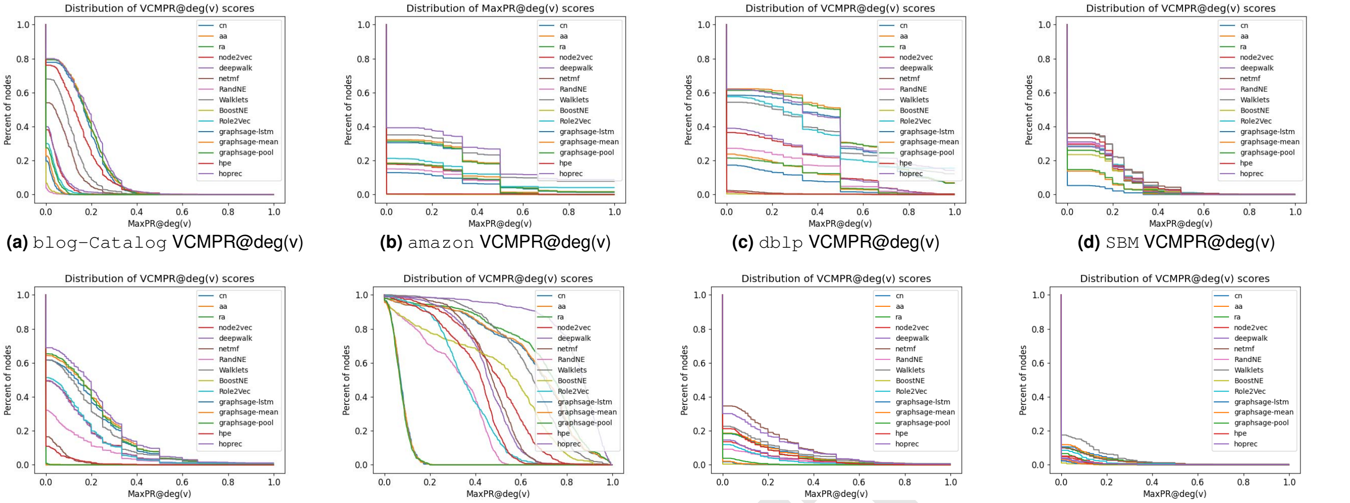

(a) `blog-Catalog` VCMPR@deg(v) **(b)** `amazon` VCMPR@deg(v) **(c)** `dblp` VCMPR@deg(v) **(d)** `SBM` VCMPR@deg(v)

(e) `ogbl-collab` VCMPR@deg(v) **(f)** `ogbl-ddi` VCMPR@deg(v) **(g)** `bioplex` VCMPR@deg(v) **(h)** `HI-II-14` VCMPR@deg(v)

Fig. 11. We compute VCMPR curves with a variable threshold set to the degree of each vertex v in the ground-truth graph. In the more sparse `amazon` and `dblp` data-sets, the VCMPR values are lower than in the fixed threshold plots. In the `blog-Catalog` dataset, the VCMPR values are slightly higher.

DRAFT

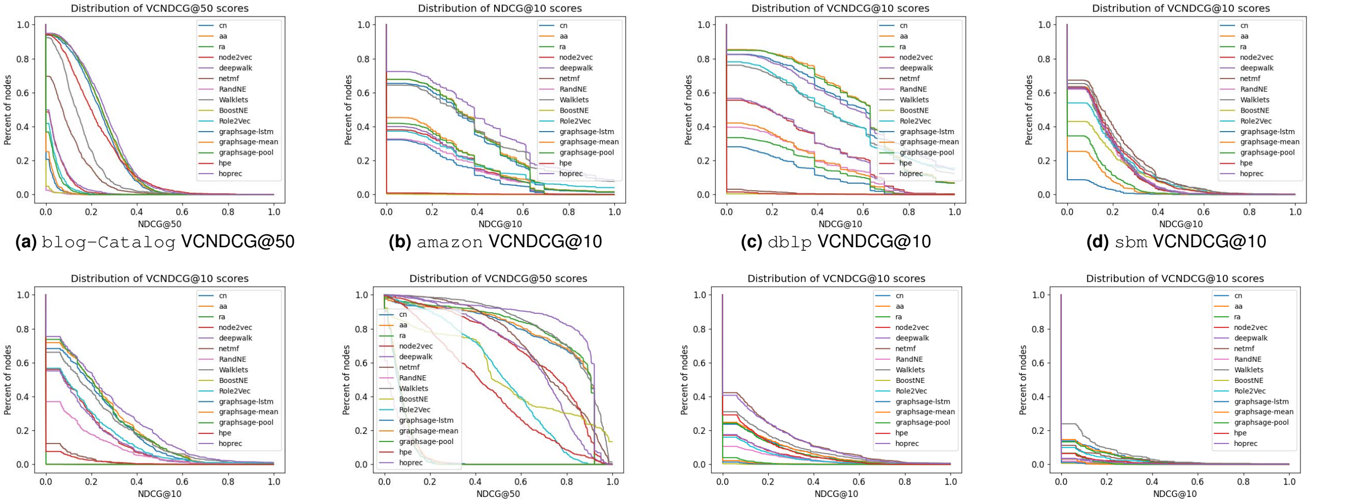

(a) `blog-Catalog` VCNDCG@50 **(b)** `amazon` VCNDCG@10 **(c)** `dblp` VCNDCG@10 **(d)** `sbm` VCNDCG@10

(e) `ogbl-collab` VCNDCG@10 **(f)** `ogbl-ddi` VCNDCG@50 **(g)** `Bioplex` VCNDCG@10 **(h)** `HI-II-14` VCNDCG@10

Fig. 12. We compute VCNDCG for all datasets. Similarly to VCMPR, the VCNDCG scores are quite low, indicating that the top predictions are of poor quality. Thus most edges are not in the top k predictions or are not ranked highly within said predictions.

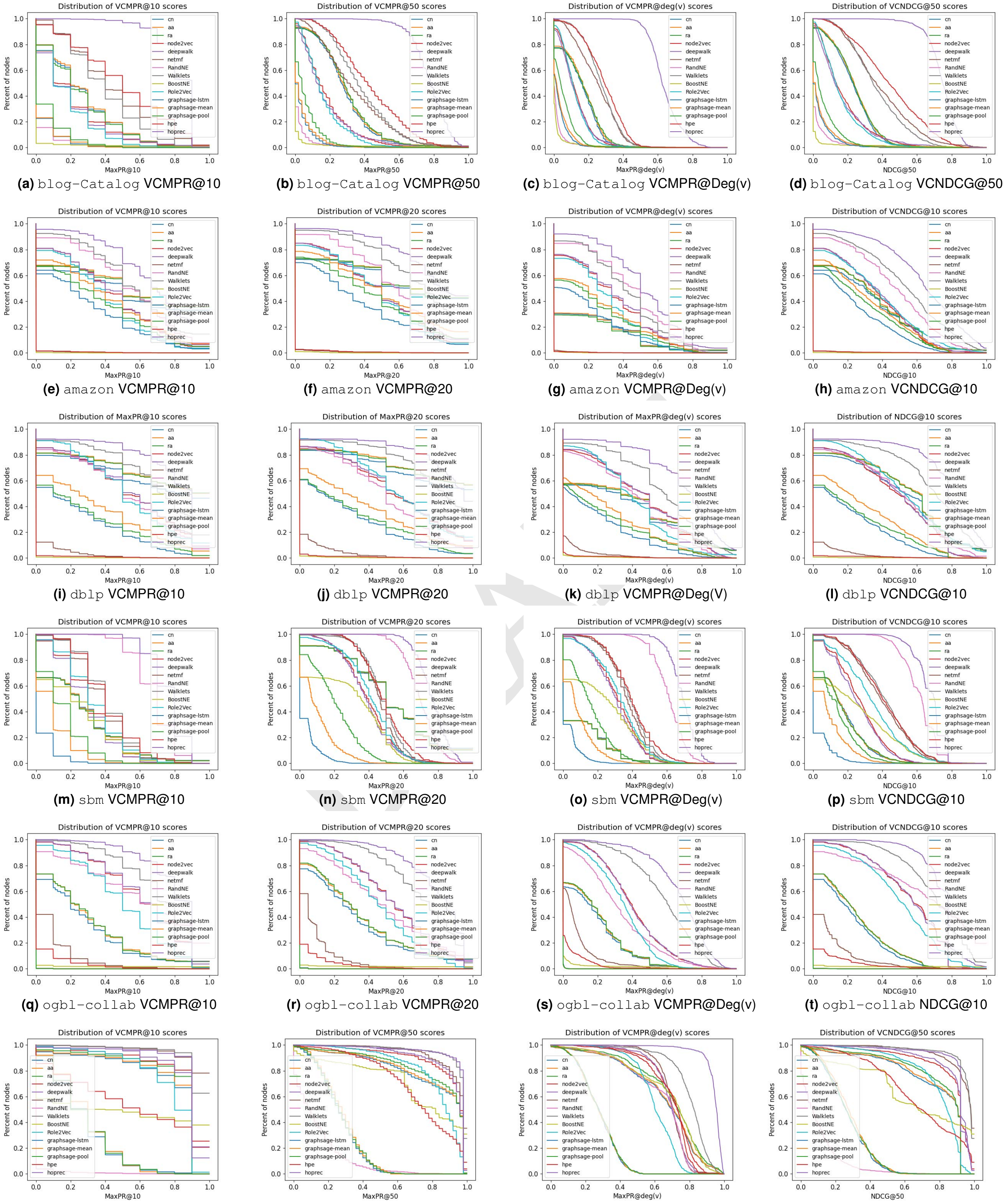

(a) `blog-Catalog` VCMPR@10 **(b)** `blog-Catalog` VCMPR@50 **(c)** `blog-Catalog` VCMPR@Deg(v) **(d)** `blog-Catalog` VCNDCG@50

(e) `amazon` VCMPR@10 **(f)** `amazon` VCMPR@20 **(g)** `amazon` VCMPR@Deg(v) **(h)** `amazon` VCNDCG@10

(i) `dblp` VCMPR@10 **(j)** `dblp` VCMPR@20 **(k)** `dblp` VCMPR@Deg(V) **(l)** `dblp` VCNDCG@10

(m) `sbm` VCMPR@10 **(n)** `sbm` VCMPR@20 **(o)** `sbm` VCMPR@Deg(v) **(p)** `sbm` VCNDCG@10

(q) `ogbl-collab` VCMPR@10 **(r)** `ogbl-collab` VCMPR@20 **(s)** `ogbl-collab` VCMPR@Deg(v) **(t)** `ogbl-collab` NDCG@10

(u) `ogbl-ddi` VCMPR@10 **(v)** `ogbl-ddi` VCMPR@50 **(w)** `ogbl-ddi` VCMPR@Deg(v) **(x)** `ogbl-ddi` VCNDCG@50

Fig. 13. VCMPR@k scores for multiple data-sets over *both the test and train edges*. Despite the fact that the majority of edges have been seen by the model in training, the VCMPR curves show that the models often do not reflect those results. `blog-Catalog` still has quite poor scores, but even for the other datasets only a small percentage of nodes have high VCMPR scores.

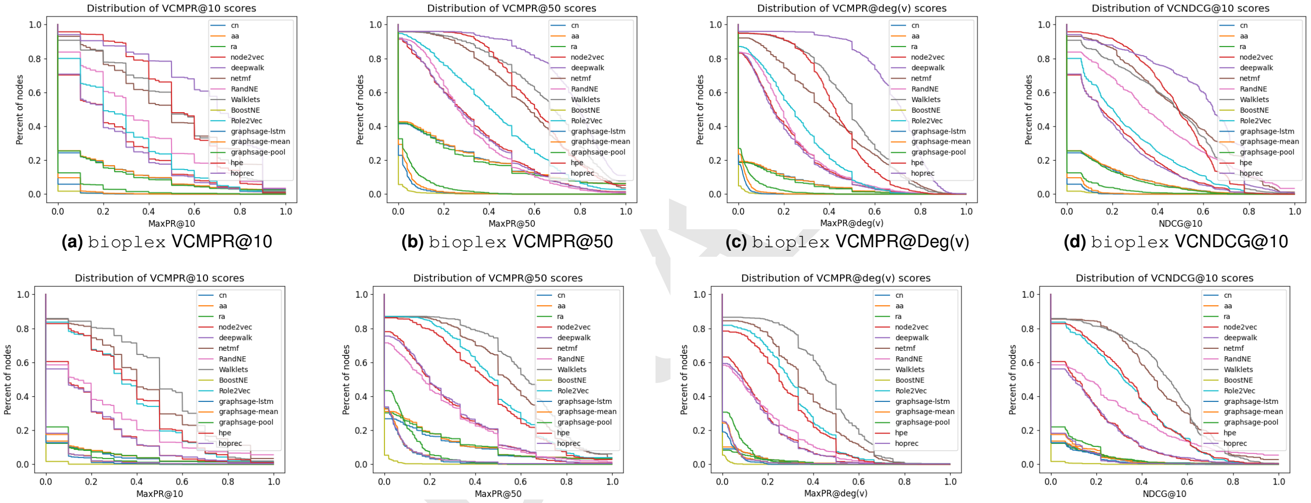

(a) `bioplex` VCMPR@10 **(b)** `bioplex` VCMPR@50 **(c)** `bioplex` VCMPR@Deg(v) **(d)** `bioplex` VCNDCG@10

(e) `HI-II-14` VCMPR@10 **(f)** `HI-II-14` VCMPR@50 **(g)** `HI-II-14` VCMPR@Deg(v) **(h)** `HI-II-14` VCNDCG@10

Fig. 14. VCMPR@k scores for PPI data-sets over *both the test and train edges*. Despite the fact that the majority of edges have been seen by the model in training, the VCMPR curves show that the models often do not reflect those results.

	Avg VCMPR @10	Avg VCMPR @50	Avg VCMPR @Deg(V)	VCNDCG @50
Common Neighbors	0.25	0.29	0.15	0.22
Adamic Adar	0.25	0.29	0.15	0.23
Resource Allocation	0.20	0.18	0.12	0.17
Deepwalk	0.19	0.17	0.12	0.16
Node2Vec	0.20	0.18	0.12	0.17
NetMF	0.43	0.34	0.25	0.38
RandNE	0.03	0.02	0.02	0.03
Walklets	0.39	0.38	0.27	0.37
BoostNE	0.01	0.01	0.01	0.01
Role2Vec	0.17	0.15	0.11	0.15
GraphSage-M	0.03	0.04	0.03	0.04
GraphSage-MP	0.06	0.06	0.05	0.06
GraphSage-L	0.03	0.03	0.03	0.03
HPE	0.49	0.43	0.29	0.44
HOP-rec	0.82	0.77	0.64	0.73

Table 13. This table complements Fig. 13, which has results from link prediction on the *entire* `blogcatalog` dataset. We give the average VCMPR@k for $k = 10, 50$, VCMPR@Deg(v), and VCNDCG@50.

	Avg VCMPR @10	Avg VCMPR @20	Avg VCMPR @Deg(V)	VCNDCG @10
Common Neighbors	0.48	0.57	0.15	0.29
Adamic Adar	0.52	0.59	0.15	0.31
Resource Allocation	0.52	0.58	0.14	0.31
Deepwalk	0.40	0.45	0.31	0.33
Node2Vec	0.42	0.47	0.31	0.34
NetMF	0.00	0.01	0.00	0.00
RandNE	0.53	0.56	0.43	0.46
Walklets	0.55	0.69	0.40	0.51
BoostNE	0.00	0.00	0.00	0.00
Role2Vec	0.37	0.45	0.28	0.36
GraphSage-M	0.38	0.47	0.25	0.29
GraphSage-MP	0.33	0.42	0.22	0.25
GraphSage-L	0.28	0.34	0.18	0.21
HPE	0.01	0.01	0.00	0.01
HOP-rec	0.67	0.78	0.86	0.63

Table 14. This table complements Fig. 13, which has results from link prediction on the *entire* `amazon` dataset. We give the average VCMPR@k for $k = 10, 20$, VCMPR@Deg(v), and VCNDCG@10.

	Avg VCMPR @10	Avg VCMPR @20	Avg VCMPR @Deg(V)	VCNDCG @10
Common Neighbors	0.64	070	0.34	0.48
Adamic Adar	0.68	0.73	0.36	0.51
Resource Allocation	0.68	0.73	0.35	0.50
Deepwalk	0.53	0.55	0.44	0.46
Node2Vec	0.53	0.54	0.43	0.45
NetMF	0.03	0.03	0.02	0.02
RandNE	0.50	0.4	0.40	0.47
Walklets	0.67	0.75	0.55	0.65
BoostNE	0.00	0.00	0.00	0.00
Role2Vec	0.52	0.57	0.39	0.51
GraphSage-M	0.32	0.35	0.24	0.27
GraphSage-MP	0.26	0.28	0.21	0.22
GraphSage-L	0.23	0.25	0.17	0.19
HPE	0.01	0.01	0.01	0.01
HOP-rec	0.77	0.82	0.66	0.74

Table 15. This table complements Fig. 13, which has results from link prediction on the *entire* `dblp` dataset. We give the average VCMPR@k for $k = 10, 20$, VCMPR@Deg(v), and VCNDCG@10.

	Avg VCMPR @10	Avg VCMPR @20	Avg VCMPR @Deg(V)	VCNDCG @10
Common Neighbors	0.25	0.50	0.08	0.15
Adamic Adar	0.24	0.50	0.08	0.14
Resource Allocation	0.24	0.50	0.08	0.15
Deepwalk	0.29	0.39	0.30	0.25
Node2Vec	0.29	0.39	0.30	0.26
NetMF	0.38	0.46	0.38	0.38
GraRep	0.40	0.49	0.39	0.39
RandNE	0.67	0.70	0.60	0.60
Walklets	0.40	0.49	0.38	0.41
BoostNE	0.22	0.27	0.21	0.22
Role2Vec	0.33	0.40	0.32	0.33
GraphSage-M	0.09	0.10	0.08	0.08
GraphSage-MP	0.14	0.19	0.15	0.12
GraphSage-L	0.03	0.03	0.03	0.03
HPE	0.41	0.48	0.39	0.40
HOP-rec	0.76	0.76	0.69	0.68

Table 16. This table complements Fig. 13, which has results from link prediction on the *entire* `sbm` dataset. We give the average VCMPR@k for $k = 10, 20$, VCMPR@Deg(v), and VCNDCG@10.

	Avg VCMPR @10	Avg VCMPR @20	Avg VCMPR @Deg(V)	VCNDCG @10
Common Neighbor	0.27	0.31	0.17	0.22
Adamic Adar	0.30	0.34	0.19	0.24
Resource Allocation	0.30	0.34	0.19	0.24
Deepwalk	0.62	0.56	0.40	0.56
Node2Vec	0.62	0.56	0.41	0.56
NetMF	0.08	0.08	0.07	0.08
RandNE	0.61	0.50	0.33	0.65
Walklets	0.71	0.71	0.58	0.62
BoostNE	0.02	0.02	0.02	0.02
Role2Vec	0.50	0.48	0.34	0.49
GraphSage-M	0.00	0.00	0.00	0.00
GraphSage-MP	0.00	0.00	0.00	0.00
GraphSage-L	0.00	0.00	0.00	0.00
HPE	0.04	0.03	0.03	0.04
HOP-rec	0.79	0.79	0.67	0.74

Table 17. This table complements Fig. 13, which has results from link prediction on the *entire* `collab` dataset. We give the average VCMPR@k for $k = 10, 20$, VCMPR@Deg(v), and VCNDCG@10.

	Avg VCMPR @10	Avg VCMPR @50	Avg VCMPR @Deg(V)	VCNDCG @50
Common Neighbors	0.76	0.81	0.64	0.76
Adamic Adar	0.77	0.82	0.65	0.77
Resource Allocation	0.79	0.83	0.68	0.79
Deepwalk	0.86	0.90	0.68	0.88
Node2Vec	0.84	0.89	0.70	0.86
NetMF	0.95	0.92	0.69	0.93
RandNE	0.01	0.12	0.62	0.60
Walklets	0.94	0.95	0.80	0.95
BoostNE	0.47	0.70	0.66	0.65
Role2Vec	0.76	0.83	0.60	0.79
GraphSage-M	0.27	0.27	0.27	0.27
GraphSage-MP	0.28	0.28	0.27	0.28
GraphSage-L	0.27	0.27	0.27	0.27
HPE	0.55	0.72	0.68	0.67
HOP-rec	0.86	0.94	0.94	0.90

Table 18. This table complements Fig. 13, which has results from link prediction on the *entire* `ddi` dataset. We give the average VCMPR@k for $k = 10, 50$, VCMPR@Deg(v), and VCNDCG@50.

DRAFT

	Avg VCMPR @10	Avg VCMPR @50	Avg VCMPR @Deg(V)	VCNDCG @10
Common Neighbors	0.10	0.18	0.05	0.07
Adamic Adar	0.10	0.19	0.05	0.07
Resource Allocation	0.09	0.18	0.05	0.07
Deepwalk	0.23	0.31	0.19	0.20
Node2Vec	0.23	0.31	0.19	0.20
NetMF	0.49	0.57	0.40	0.49
RandNE	0.36	0.31	0.22	0.39
Walklets	0.49	0.64	0.48	0.45
BoostNE	0.00	0.00	0.00	0.00
Role2Vec	0.28	0.42	0.25	0.25
GraphSage-M	0.01	0.02	0.01	0.01
GraphSage-MP	0.02	0.03	0.02	0.02
GraphSage-L	0.01	0.01	0.01	0.01
HPE	0.53	0.60	0.42	0.47
HOP-rec	0.64	0.74	0.67	0.57

Table 19. This table complements Fig. 14, which has results from link prediction on the *entire* `Bioplex` dataset. We give the average VCMPR@k for $k = 10, 50$, VCMPR@Deg(v), and VCNDCG@10.

	Avg VCMPR @10	Avg VCMPR @50	Avg VCMPR @Deg(V)	VCNDCG @10
Common Neighbors	0.04	0.10	0.02	0.03
Adamic Adar	0.05	0.11	0.02	0.03
Resource Allocation	0.04	0.11	0.02	0.03
Deepwalk	0.17	0.25	0.13	0.16
Node2Vec	0.17	0.25	0.14	0.16
NetMF	0.42	0.52	0.34	0.45
RandNE	0.23	0.23	0.14	0.25
Walklets	0.48	0.57	0.42	0.46
BoostNE	0.00	0.00	0.00	0.00
Role2Vec	0.35	0.45	0.28	0.32
GraphSage-M	0.03	0.04	0.02	0.03
GraphSage-MP	0.04	0.06	0.03	0.04
GraphSage-L	0.02	0.03	0.01	0.02
HPE	0.36	0.42	0.26	0.33
HOP-rec	0.03	0.04	0.02	0.03

Table 20. This table complements Fig. 14, which has results from link prediction on the *entire* `HI-II-14` dataset. We give the average VCMPR@k for $k = 10, 50$, VCMPR@Deg(v), and VCNDCG@10.

DRAFT
